# Defensins as natural antimicrobial peptide scaffolds against antimicrobial-resistant pathogens: mechanisms, resistance risks, and translational prospects

**DOI:** 10.3389/fcimb.2026.1881716

**Published:** 2026-08-24

**Authors:** Emad M. Abdallah, Samiah Hamad Al-Mijalli, Nawal Al Hakawati, Alissar Al Khatib

**Affiliations:** 1 Department of Biology, College of Science, Qassim University, Buraydah, Saudi Arabia; 2 Department of Biology, College of Sciences, Princess Nourah bint Abdulrahman University, Riyadh, Saudi Arabia; 3 Department of Biological Sciences, Faculty of Science, Beirut Arab University, Tripoli, Lebanon; 4 Department of General Studies, Almoosa College of Health Sciences, Al Ahsaa, Saudi Arabia

**Keywords:** antimicrobial peptides, biofilms, drug delivery, drug development, infectious disease, multidrug resistance, peptidomimetics

## Abstract

The growing threat of antimicrobial resistance has increased the need for anti-infective approaches beyond conventional single-target antibiotics. Defensins, a conserved family of cysteine-rich antimicrobial peptides, are promising candidates owing to their structural stability, membrane activity, target-specific mechanisms, immunomodulatory functions, and potential synergy with existing antibiotics. This critical narrative review discusses defensins as potential therapeutics against antimicrobial-resistant pathogens, with a focus on structure-activity relationships, bacterial envelope biology, resistance evolution and cross-resistance, antibiofilm activity, and translational feasibility. Data were synthesized from mammalian, plant, fungal, and insect defensins, together with defensin-derived peptides and defensin mimetics. Unrelated AMPs were included only as contextual comparators and were not treated as defensin-specific evidence. Antibacterial proof-of-concept and translational-readiness evidence were appraised separately, including activity under physiological ionic-strength and serum conditions, protease stability, cytotoxicity, hemolysis, resistance selection, and *in vivo* efficacy. However, clinical translation has been hampered by inconsistent testing standards, incomplete pharmacokinetic/pharmacodynamic characterization, safety concerns, manufacturing challenges, and inadequate resistance surveillance. Existing evidence does not support classifying defensins as resistance-proof or uniformly less resistance-prone than conventional antibiotics. Instead, they should be regarded as versatile scaffolds whose resistance risk requires candidate-specific evaluation. Future studies should combine standardized broth microdilution with testing in serum, protease-rich environments, and mature biofilms. Candidate progression should require infection-site PK/PD, route-appropriate safety, and efficacy in chronic-wound, device-biofilm, or mucosal-infection models. AI-guided design and delivery systems should advance only when they demonstrably improve stability, exposure, activity, or tolerability, with resistance monitored throughout development and use.

## 1 Introduction

Antimicrobial resistance (AMR) has become one of the greatest challenges facing modern medicine and public health worldwide. The rapid emergence and spread of multidrug-resistant organisms have pushed this problem to a critical global level. What was once an early warning in the beginning of the antibiotic era is now a global reality, with resistant infections causing more than 1.27 million deaths annually (Marino et al., 2025). Moreover, AMR has been projected to cause up to 10 million deaths annually by 2050, with cumulative economic losses approaching USD 100 trillion (O’Neill, 2016). This projected burden has intensified efforts to identify and develop alternative antimicrobial strategies, including natural products and antimicrobial peptides. However, natural products and medicinal plants continue to face substantial barriers to translation into clinically useful antimicrobials, including the predominance of *in vitro* evidence, limited mechanistic and safety data, poor standardization of antibacterial testing, absence of agreed interpretive cutoffs, weak progression from initial screening to active-compound isolation, the high cost and prolonged timeline of drug development, and pronounced phytochemical variability driven by seasonal and environmental factors (Abdallah et al., 2023). Natural Antimicrobial peptides (AMPs) are small bioactive molecules widely distributed across diverse life forms, including archaea, bacteria, fungi, protists, plants, and mammals. According to the 2024 update of the AMP database, approximately 3,940 antimicrobial peptides have been documented across six biological kingdoms (Zhao et al., 2024). AMPs are especially attractive in early drug discovery because their structures are well defined, can be modified by design, and are increasingly supported by computational tools. However, their clinical use is still limited by important challenges, including instability, delivery difficulties, possible toxicity, and high production costs (Yang et al., 2025). Defensins are a prominent family of small, cationic, cysteine-rich antimicrobial peptides that constitute an important component of innate immunity across diverse organisms and act as rapid first-line effectors against pathogens such as bacteria, fungi, and viruses (Ganz, 2003; Jarczak et al., 2013). Defensins exhibit mechanistic diversity that may support activity against pathogens refractory to classical therapies (Pimentel et al., 2026). However, mechanistic multiplicity does not by itself establish a reduced probability of resistance development. Comparative experimental evidence indicates that resistance outcomes are peptide-, pathogen-, and protocol-dependent. In *Escherichia coli*, resistance gains across 14 tested antimicrobial peptides were lower on average, but substantially more heterogeneous, than those observed across 12 antibiotics, with several peptides nevertheless permitting appreciable resistance evolution (Spohn et al., 2019). Conversely, serial exposure of *Staphylococcus aureus* to host-defense peptides selected stable resistance with little or no detectable fitness cost and reduced susceptibility to human defense peptides and clinically used antibiotics (Kubicek-Sutherland et al., 2017). Bacteria can decrease peptide susceptibility through membrane and cell-envelope remodeling, proteolytic degradation, efflux, electrostatic repulsion, peptide trapping, and coordinated stress responses (Tajer et al., 2024). Therefore, defensins should not be assigned a class-wide resistance advantage; resistance liability must be established for each peptide–pathogen combination under defined exposure and physiological conditions. Despite their biological promise, the clinical translation of defensins has been constrained by well-recognized pharmacological barriers. These include susceptibility to proteolytic degradation, limited bioavailability, potential cytotoxicity at higher concentrations, and challenges in large-scale manufacturing and delivery (Zhao et al., 2024). Consequently, the field has shifted toward engineering strategies that retain the antimicrobial core of defensins while overcoming these limitations. These approaches include peptide truncation, cyclization, amino acid substitution, nano-delivery systems, and the development of non-peptidic mimetics that replicate key physicochemical features such as amphipathicity and charge distribution (Ali et al., 2025). Parallel advances in computational design and artificial intelligence are accelerating the identification of optimized peptide analogues with enhanced stability, selectivity, and reduced toxicity, marking a transition from natural templates to rationally designed therapeutics (Wang et al., 2025). Another critical dimension driving renewed interest in defensins is their capacity for synergistic interaction with existing antibiotics. Rather than replacing conventional drugs, defensins can restore antibiotic efficacy, disrupt biofilms, and reduce required dosing, thereby contributing to resistance containment strategies rather than acting as standalone solutions (Roque-Borda et al., 2026). This reflects a broader transition in AMR research away from single-agent “silver bullet” approaches toward combinatorial, mechanism-informed, and systems-level therapeutic strategies.

Although interest in defensins as anti-MDR candidates continues to grow, the field remains fragmented. Many reviews describe defensins broadly within the AMP literature, but few critically connect defensin structure, pathogen-envelope biology, resistance adaptations, and translational feasibility in one framework (Gao et al., 2021; Fu et al., 2023). In addition, much of the reported antibacterial evidence is still derived from simplified *in vitro* assays that do not adequately reflect physiological salt concentrations, serum binding, protease exposure, biofilm conditions, or pathogen-specific envelope barriers (Grassi et al., 2019; Yao et al., 2025). As a result, it remains unclear which defensin scaffolds are genuinely promising for MDR infections, under which biological conditions they retain activity, and where engineered analogues or mimetics offer realistic therapeutic advantages (Luo et al., 2022). This gap limits rational prioritization of defensin-based candidates for clinical development, particularly because bacterial resistance to AMPs can involve surface remodeling, proteolysis, efflux, and reduced electrostatic attraction (Maria-Neto et al., 2015).

This critical narrative review aimed to evaluate defensins as candidate therapeutics against AMR pathogens. It integrates structural biology with functional microbiology by outlining major defensin classes and identifying the physicochemical features that determine activity under physiologically relevant conditions. The review critically assesses current anti-AMR evidence, prioritizing studies that account for ionic strength, serum effects, and protease susceptibility. It further maps defensin mechanisms of action to bacterial envelope biology, linking peptide behavior to pathogen-specific vulnerabilities and resistance adaptations. Evidence is evaluated across priority AMR pathogens within relevant infection contexts. Finally, the review examines translational progress, including engineered analogues, mimetics, and delivery strategies, and proposes a practical roadmap that emphasizes standardization and future directions.

## 2 Methodological approach

This article was conducted as a critical narrative review. A broad, non-exhaustive literature survey was undertaken using PubMed/MEDLINE, Scopus, Web of Science, ScienceDirect, and Google Scholar. The review focused mainly on publications from 2016 to 2026, while earlier landmark studies were included when required for mechanistic or historical context. Search concepts included defensins, antimicrobial peptides, antimicrobial resistance, AMR and ESKAPE pathogens, biofilms, resistance and cross-resistance, peptide engineering, peptidomimetics, and drug delivery. Publications were selected purposively according to their relevance to defensin structure and activity, bacterial-envelope interactions, mechanisms of action, resistance, antibiofilm effects, physiological stability, safety, engineering, delivery, and translational development. Greater interpretive weight was assigned to experimental studies that characterized both the peptide and bacterial strain and reported standardized microbiological, mechanistic, stability, or toxicity outcomes. For the representative experimental studies summarized in **Table 1**, we recorded whether salt/ionic effects, serum effects, protease stability, cytotoxicity, hemolysis, resistance-selection testing, and *in vivo* efficacy were directly assessed; unreported endpoints were designated NR and were not inferred from other publications. To preserve the defensin-focused scope, candidates were classified as native defensins, defensin-derived or engineered analogues retaining an identifiable defensin scaffold, defensin-inspired mimetics, or unrelated natural or engineered AMPs used solely as comparators. Comparator evidence, including melittin, was included only to illustrate general mechanistic or translational principles relevant to defensin research and was not extrapolated to defensin-specific mechanisms, efficacy, safety, or translational potential. These categories were distinguished consistently throughout the text, figures, and tables. Purely computational studies were treated as supportive unless accompanied by experimental validation. Because this article was designed as a structured narrative review rather than a formal systematic review, no PRISMA flow diagram, preregistered protocol, or quantitative record accounting was undertaken.

**Table 1:** Antibacterial and translational-readiness evidence for defensins, related candidates, mimetics, and comparator AMPs.

Author(s)	Candidate compound(s) and classification	Antibacterial evidence	Salt/ionic effects	Serum effects	Protease stability	Cytotoxicity	Hemolysis	Resistance selection	*In vivo* evidence
Jekhmane et al. (2024)	Plectasin — native fungal defensin	Lipid-II recognition and Ca²^+^-enhanced assembly in Gram-positive bacteria.	Ca²^+^/Mg²^+^ effects assessed; physiological NaCl not tested.	NR	NR	NR	NR	NR	NR
Luo et al. (2022)	Compound 10 — HD5-inspired defensin mimetic	Activity against MDR/PDR isolates; OmpA/LPS binding, membrane disruption, and ribosomal inhibition.	Mg²^+^ effects assessed; physiological-salt panel not reported.	NR	NR	Low toxicity; CC_50_ >20 μM.	NR	NR	Murine septicemia and pneumonia models; plasma half-life 2.35 h.
Shen et al. (2025)	Peptide 4 — unrelated microbiome-derived AMP comparator	Anti-MRSA activity and membrane disruption.	NR	NR	NR	Low toxicity in intestinal epithelial cells; skin tolerability assessed.	<15% at 32× MIC in mouse and bovine erythrocytes.	No MIC increase after 26-day serial passage.	Murine MRSA wound model.
Yadav et al. (2025)	d-WRL and W(Dab)L — unrelated engineered AMP comparators	Anti-MRSA and antibiofilm activity through membrane disruption.	Activity retained under tested physiological-salt conditions.	Stable in human serum for up to 6 h.	Stable after protease exposure.	Low toxicity in tested human cell lines.	Minimal; none detected at the MIC.	No resistance after 30 days/96 generations.	NR
Escobar-Salom et al. (2024)	HNP-1 and hBD-3 — native human defensins	Activity against MDR Gram-negative ESKAPE isolates; hBD-3 showed species-dependent efficacy.	NR	NR	NR	NR	NR	NR	NR
Gbala et al. (2022)	Defensin-d2 and actifensin — plant defensin and bacterial defensin-like AMP	Antibacterial, antifungal, membrane-disruptive, and antibiofilm activities.	NR	NR	NR	NR	<3% in mouse erythrocytes.	Organism-dependent MIC increases during serial passage; no change in *C. albicans*.	NR
Mirzaei et al. (2023)	Melittin — unrelated non-defensin AMP comparator	Antibiofilm activity and antibiotic synergy against MDR-MRSA and MDR *P. aeruginosa*.	NR	NR	NR	Concentration-dependent HEK-293 toxicity; none at synergistic concentrations.	Concentration-dependent; none at synergistic concentrations.	NR	NR
Bolatchiev (2022)	hBD-3 and epinecidin-1 — native defensin and unrelated fish AMP comparator	Activity and antibiotic interactions against resistant clinical isolates.	NR	NR	NR	NR	NR	NR	hBD-3 and its antibiotic combinations evaluated in murine sepsis models.
Abdelsattar et al. (2025)	Brilacidin — synthetic defensin mimetic	Activity against MDR *N. gonorrhoeae* through membrane disruption and rapid killing.	NR	NR	NR	High ME-180 cell viability up to 64 μg/mL.	HC₉_0_ >128 μg/mL.	NR	NR

*NR, not reported in the cited study; NR denotes an evidence gap and should not be interpreted as an unfavorable outcome.

## 3 Defensins as antimicrobial scaffolds: structural identity and therapeutic logic

Defensins are a conserved subgroup of antimicrobial peptides characterized by short length, net cationic charge, cysteine-rich sequences, and conserved intramolecular disulfide bonds, features that distinguish them structurally from many flexible linear AMPs (Gao et al., 2021). Defensins are not a single uniform group, but conserved peptide scaffolds found in mammals, plants, and invertebrates. Their main subclasses α-, β-, and θ-defensins differ in disulfide bonding and overall topology, which can affect target recognition, membrane binding, and pharmacological behavior. Thus, defensins should be viewed as distinct structural frameworks, not interchangeable AMPs (Zhao et al., 2024). The key structural and functional distinctions between defensins and other major AMP classes are summarized in **Table 2**, emphasizing the scaffold features that support their therapeutic engineering.

**Table 2:** Comparative structural and translational features of defensins and major antimicrobial peptide classes.

AMP class	Structural determinants	Physiological stability	Major mechanism(s)	Primary limitation	References
Defensins	Disulfide-constrained (6 Cys)	Protease-stable; salt/serum sensitive	Multi-modal (membrane, lipid II, enzymatic, immune)	Ionic sensitivity; delivery	(Ganz, 2003)
Linear α-helical AMPs	Flexible amphipathic helix	Protease/serum labile	Membrane permeabilization	Low *in vivo* stability; toxicity	(Xuan et al., 2023)
Cyclic/constrained peptides	Backbone-cyclized/rigid	Protease-resistant; serum-stable	Membrane or target-specific	Synthetic complexity	(Luo et al., 2022)
Lipopeptides	Lipid–peptide conjugate	Strong membrane binding	Membrane depolarization	Toxicity; resistance adaptation	(Xuan et al., 2023)
Defensin mimetics	Synthetic constrained scaffold	PK-optimized	Tunable (membrane/target)	Safety/selectivity validation	(Luo et al., 2022)

For therapeutic development, defensins are better viewed not as simple “natural antibiotics,” but as structured scaffolds that can be engineered. Their activity depends on several linked features: net charge and charge distribution guide interaction with microbial surfaces, but stronger cationicity does not always mean better selectivity and may increase toxicity or serum binding (Xuan et al., 2023). Their disulfide-stabilized fold supports structural stability and protease resistance, helping distinguish them from many linear AMPs. In some defensins, higher-order assembly is also functionally important, as shown in plectasin, where supramolecular organization contributes to lipid II targeting and antibacterial action (Jekhmane et al., 2024). Functionally, defensins extend beyond simple membrane disruption. Although electrostatic interaction with microbial membranes is often the initial step, certain defensins inhibit cell-wall biosynthesis through specific molecular interactions; for example, plectasin binds directly to lipid II (Jekhmane et al., 2024). In addition, defensins exhibit well-documented immunomodulatory roles, including regulation of inflammatory signaling and innate immune activation (Nagib et al., 2025). This mechanistic diversity distinguishes defensins from single-target antibiotics and positions them as multifunctional host-defense effectors rather than simple antimicrobial peptides.

This mechanistic breadth does not, by itself, establish either reduced resistance risk or therapeutic feasibility; both require candidate-specific experimental assessment. MIC/MBC values, time–kill kinetics, membrane disruption, antibiofilm activity, and activity against MDR isolates establish antibacterial proof of concept. Translational readiness additionally requires retained activity under physiological salt and serum conditions, protease stability, acceptable cytotoxicity and hemolysis, low resistance-selection liability, and efficacy in an appropriate *in vivo* infection model (Mookherjee et al., 2020; Zheng et al., 2025). Accordingly, the studies discussed below are interpreted only at the level supported by their reported endpoints, and untested variables are not inferred. Therapeutically, defensins sit at the interface between natural host defense and rational drug design. Although native peptides may face challenges in stability, delivery, and production, their conserved scaffolds can be redesigned through truncation, cyclization, or peptidomimetic chemistry. Defensins are therefore better viewed as templates for next-generation anti-infectives rather than fixed natural products, particularly for MDR pathogens (Luo et al., 2022).

## 4 Defensin classes and structural diversity

**Figure 1:**
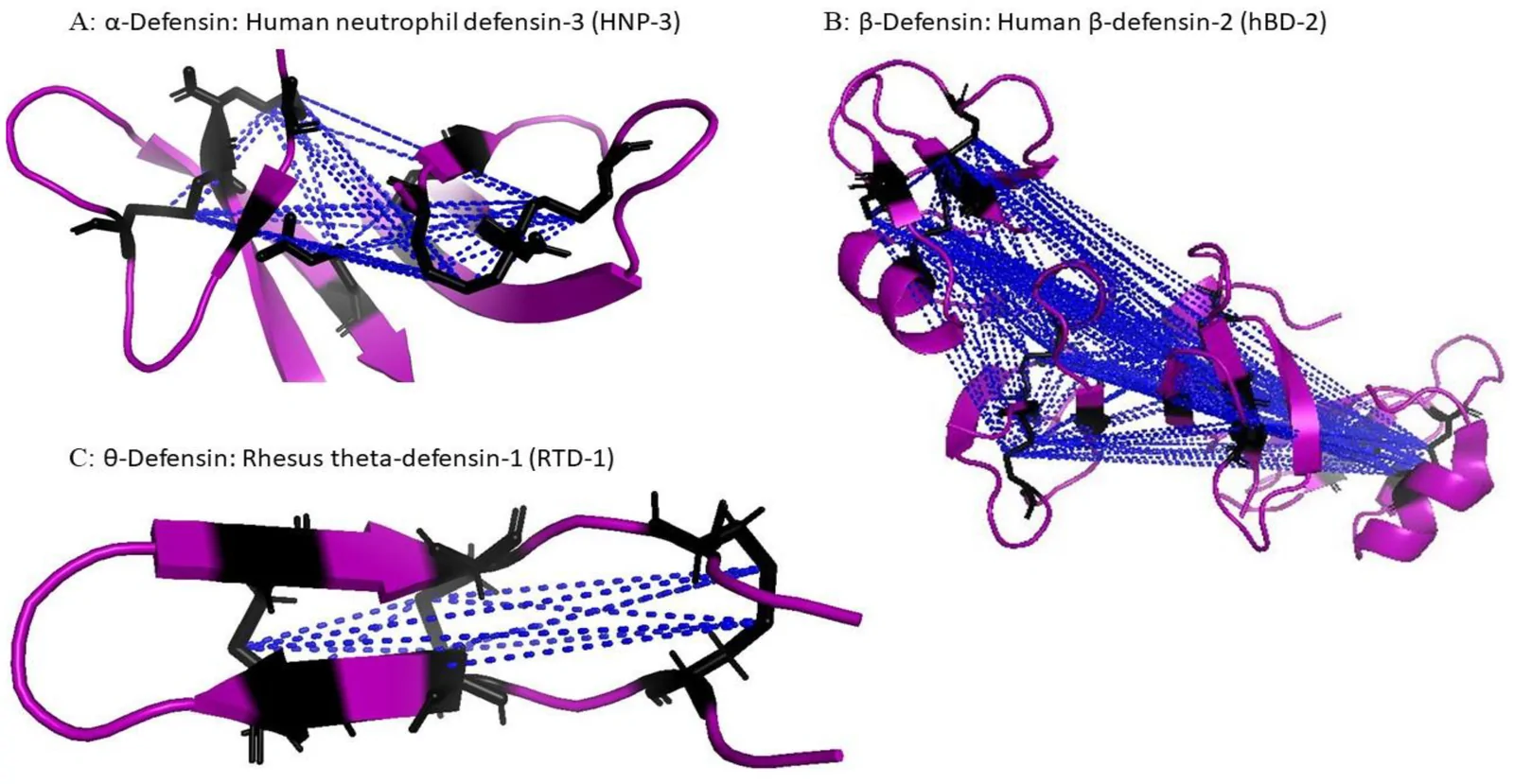
Structure of defensin families. Structures of the three major classes of defensins are represented by **(A)** α-defensins (HNP-3), **(B)** β-defensins (hBD-2), and **(C)** θ-defensins (RTD-1). All defensins exhibit antimicrobial activity, however, they have different peptide structures and patterns of disulfide bonding. These differences contribute to the differences in stability and how they interact with microbial membranes. The cysteine residues are depicted in black and the disulfide bonds in blue.

Defensins are defined as a conserved superfamily of cysteine-rich host defense peptides that exist in vertebrates, invertebrates, plants, and even microbial ecosystems (Fu et al., 2023; Zhao et al., 2024). These proteins can be sorted into structurally and evolutionarily distinct subclasses which are α-, β-, and θ-defensins in mammals as well as defensin-like peptides in insects and plants (**Figure 1**) (Fu et al., 2023; Nagib et al., 2025). Each subclass has a unique peptide structure, mechanism of action, pharmacokinetics, and rational drug design against multidrug-resistant (MDR) pathogens. This uniqueness is due to the structural and evolutionary differences of these subclasses (Xuan et al., 2023; Salehi et al., 2025).

### 4.1 α-Defensins

**Figure 2:**
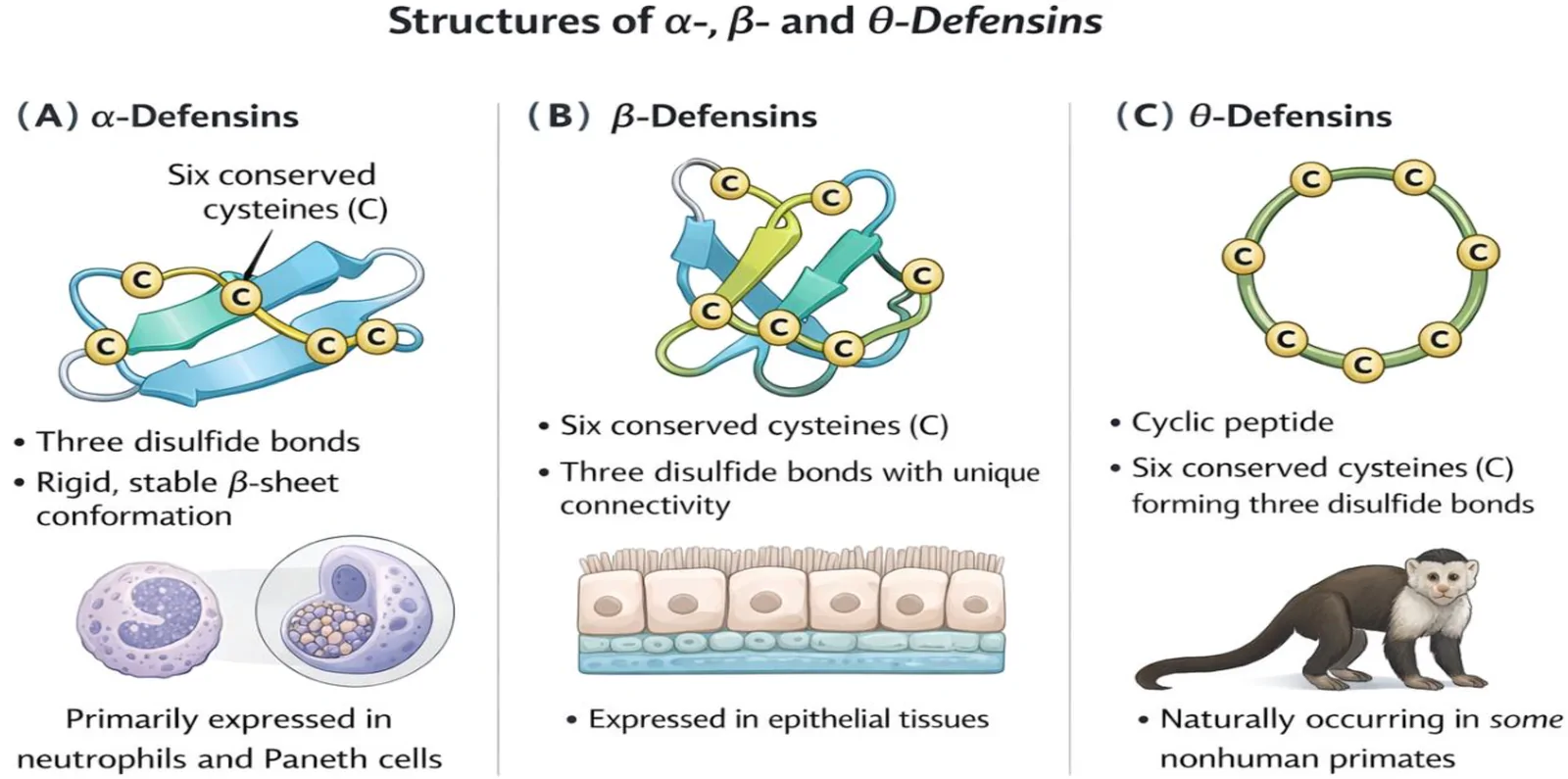
Structural diversity of α-, β-, and θ-defensins. **(A)** α-Defensins, which are mainly expressed in neutrophils and Paneth cells, have six conserved cysteines that form three disulfide bonds that stabilize a rigid β-sheet structure. **(B)** β-Defensins, which are primarily found in epithelial tissues, have a unique disulfide connectivity and loop topology despite sharing six conserved cysteines. **(C)** θ-Defensins are cyclic peptides with improved stability and protease resistance that are naturally found in some nonhuman primates. They are created by head-to-tail ligation of two α-defensin precursors. Important structural elements that affect their stability, antimicrobial activity, and potential for therapeutic design are highlighted in this schematic (Created with BioRender.com).

α-Defensins are abundant in mammals specifically primates including humans where they are primarily expressed in neutrophils and Paneth cells (**Figure 2A**) (Fu et al., 2023; Nagib et al., 2025). These proteins contain six conserved cysteines, which form three disulfide bonds with characteristic connectivity forming a rigid, stable β-sheet conformation (Zhao et al., 2024). The compactness of the disulfide-stabilized structure makes it resistant to proteolysis and preserves the protein’s integrity in protease-dense spaces such as inflamed mucosa (Fu et al., 2023). α-Defensins also have antimicrobial and immunomodulatory functions such as chemotaxis and cytokine modulation, emphasizing their “double-edged sword” role in host immunity (Xu and Lu, 2020). Therefore, therapeutic development of α-defensins requires careful optimization of their immunomodulatory activity to avoid excessive inflammation (Xu and Lu, 2020). α-defensins are capable of forming stable scaffolds as a result of their very rigid structure, however, due to their high disulfide complexity, they may present a challenge for recombinant and large-scale production (Travé-Asensio et al., 2025).

### 4.2 β-Defensins

β-Defensins mostly exist in vertebrate epithelial tissues, including skin, respiratory epithelium, and gastrointestinal mucosa (**Figure 2B**) (Fu et al., 2023; Zhao et al., 2024). They contain six conserved cysteine residues, like α-Defensins, but have a unique disulfide bond connection pattern that gives them a different loop topology and surface charge distribution different than that of α-Defensins (Nagib et al., 2025). Their epithelial localization supports their investigation for topical and mucosal applications. For instance, human β-defensin-2 (hBD-2) plays a protective role in epithelial barrier integrity in methicillin-resistant Staphylococcus aureus infection, particularly in chronic rhinosinusitis with nasal polyps (Tian et al., 2025). Moreover, smaller β-defensin-derived peptides retain activity against MDR bacterial strains through structural truncation strategies showing that minimal structural motifs can preserve antimicrobial efficacy (Colicchio et al., 2021). Although truncation may improve tissue penetration and reduce manufacturing costs, it may also alter peptide stability, target specificity, or immunomodulatory activity and therefore requires careful optimization during therapeutic development. Moreover, defensin-inspired peptidomimetics have been designed to enhance metabolic stability for improved scalability without compromising their amphipathic topology or antimicrobial activity (Luo et al., 2022). Nevertheless, important translational challenges remain, including suboptimal pharmacokinetic performance, uncertain long-term safety and immunogenicity, and the need for cost-effective large-scale manufacturing. Accordingly, the structural flexibility of β-defensins provides a useful framework for redesign, but further preclinical and clinical evaluation is required to establish their efficacy against MDR infections (Xuan et al., 2023; Travé-Asensio et al., 2025).

### 4.3 θ-Defensins

θ-Defensins are cyclic peptides generated by head-to-tail ligation of two truncated α-defensin-related precursor peptides. While they occur naturally in some nonhuman primates, the corresponding human genes contain premature stop codons that prevent the production of mature θ-defensins (**Figure 2C**) (Fu et al., 2023; Nagib et al., 2025). Cyclic backbones provide greater resistance to proteolytic breakdown, conferring greater proteolytic stability than that of comparable linear peptides (Nagib et al., 2025). Cyclical backbones are therefore an important tool for designing peptide therapeutics because they enhance both stability of circulating peptides and overall bioavailability of these drugs (Xuan et al., 2023). Applying cyclization or related conformational constraints to α- or β-defensin derivatives, it is possible to enhance both the pharmacokinetics and longevity of peptides *in vivo* (Salehi et al., 2025).

### 4.4 Evolutionary diversification beyond mammals

Defensin-like peptides can be found in various organisms including insects, plants and mammals, however as previously mentioned their shapes and structures are very different in nature. A new category of defensin-like peptides derived from Black soldier fly (*Hermetia illucens*) exhibit antibacterial activity, showing that defensin scaffolds have evolved to function in invertebrate species (Fahmy et al., 2024). Also, plant defensins exhibit antibacterial properties against *Pseudomonas* species through membrane interactions and intracellular effects, suggesting a mechanistic diversity that is distinct from mammalian defensins (Sathoff et al., 2020). Furthermore, deep learning-based analysis of ruminant gastrointestinal microbiomes has produced new anti-MRSA AMP candidates, illustrating that evolutionary biodiversity can be utilized for antibiotic discovery (Shen et al., 2025).

### 4.5 Structure-mechanism relationships and target specificity

**Figure 3:**
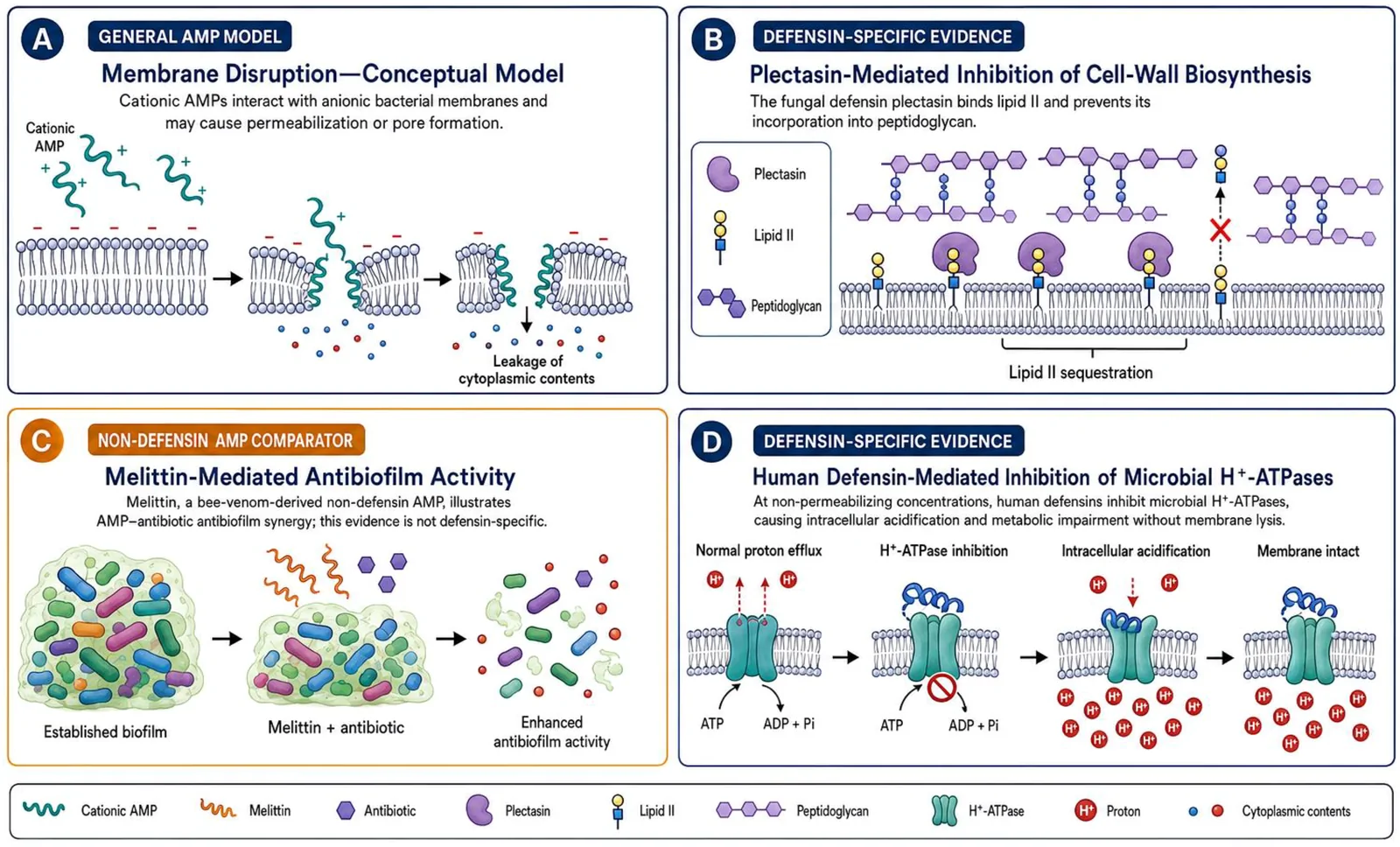
Defensin-specific mechanisms and contextual AMP comparators. **(B, D)** present defensin-specific evidence, whereas **(A, C)** provide contextual comparisons and do not represent universal defensin behavior. **(A)** General AMP-mediated membrane permeabilization and cytoplasmic leakage. **(B)** Plectasin binding and sequestration of lipid II, inhibiting cell-wall biosynthesis. **(C)** Melittin, an unrelated non-defensin AMP, illustrates AMP–antibiotic antibiofilm synergy; this evidence is not extrapolated to defensins. **(D)** Human defensin-mediated inhibition of microbial H^+^-ATPases, causing intracellular acidification and metabolic impairment without membrane lysis.

Defensins have historically been characterized as membrane-disrupting peptides that cause membrane rupture and ion leakage; however, numerous studies have revealed more complex mechanisms (**Figure 3A**). Plectasin, a fungal defensin peptide with potent, targeted antibiotic activity against Gram-positive bacteria, stops the synthesis of the cell wall instead of breaking down the membrane (Jekhmane et al., 2024). Plectasin first binds the bacterial cell wall precursor lipid II with high specificity. Calcium subsequently promotes the formation of supramolecular plectasin-lipid II assemblies, leading to peptide oligomerization and stabilization of the complex through interactions with the lipid II pyrophosphate moiety. The resulting sequestration of lipid II prevents its incorporation into the growing peptidoglycan layer, thereby inhibiting bacterial cell wall biosynthesis rather than disrupting membrane integrity (**Figure 3B**). For contextual comparison, melittin, a bee-venom-derived non-defensin AMP, is included solely to illustrate AMP–antibiotic antibiofilm synergy. In MDR-MRSA and *P. aeruginosa* models, melittin inhibited biofilm formation and enhanced activity against established biofilms when combined with conventional antibiotics (**Figure 3C**) (Mirzaei et al., 2023). Because melittin is structurally unrelated to defensins, these findings are not interpreted as evidence of equivalent mechanisms or efficacy in defensins, demonstrated prevention of biofilm formation and eradication of established biofilms against many multidrug-resistant (MDR) species, such as *S. aureus* and *P. aeruginosa*, particularly when combined with established antibiotics (**Figure 3C**) (Mirzaei et al., 2023). Furthermore, human defensins can inhibit microbial plasma membrane H^+^ ATPases instead of directly lysing membranes, at physiological, non-permeabilizing concentrations, showing a mechanism of action that extends beyond pore formation (**Figure 3D**) (Andrés et al., 2024).

The existence of multiple structural classes of defensins (α-β-θ) and the presence of insect-, plant-, and microbiome-derived peptides that share similarities to defensins, provide a broad evolutionary basis for creating new classes of antimicrobial drugs (Fu et al., 2023). Disulfide architecture influences folding stability and protease resistance, cyclic backbones improve pharmacokinetic durability, and loop topology determines target specificity and salt tolerance (Nagib et al., 2025; Travé-Asensio et al., 2025). Understanding the different structural classes of defensins proves crucial not only in evolutionary biology, but also for the development of rationally designed peptides that could act as drugs through enhanced delivery methods or chemical modifications. Studies examining charge density, amphipathicity, hydrophobic moment, structural constraint, and salt tolerance reveal these characteristics significantly affect how effective a compound is against MDR organisms (Xuan et al., 2023; Salehi et al., 2025; Travé-Asensio et al., 2025) New drugs to combat infections resistant to many currently available drugs will also be developed as a result of this information (Salehi et al., 2025).

## 5 Structure–activity drivers of anti-MDR efficacy

**Figure 4:**
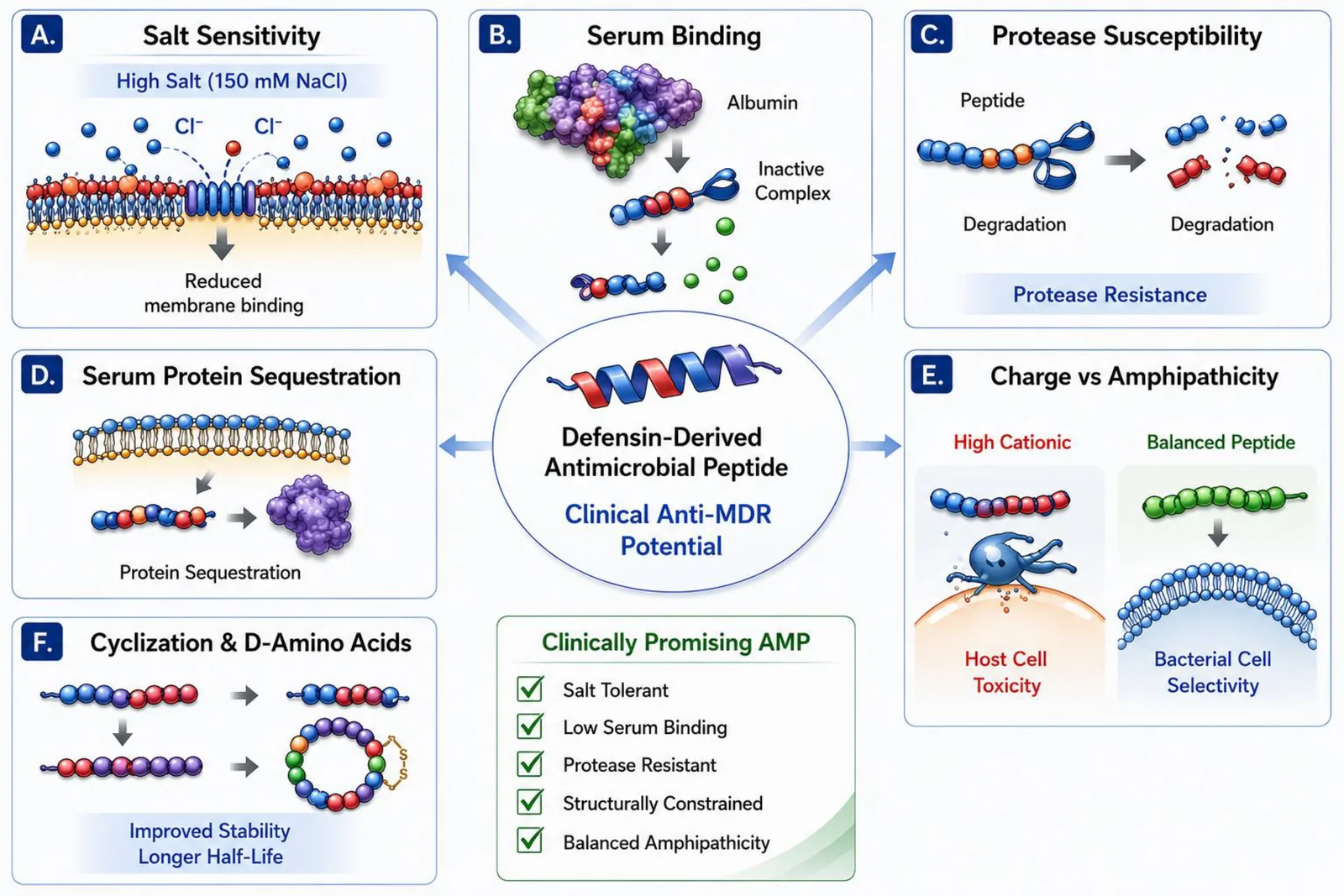
Key determinants of antimicrobial peptide performance against MDR pathogens. The figure summarizes major factors that shape peptide activity under physiological conditions, including **(A)** salt sensitivity and reduced membrane binding at high ionic strength; **(B)** serum binding and peptide inactivation; **(C)** susceptibility to proteolytic degradation; **(D)** sequestration by serum proteins; **(E)** the balance between cationic charge and amphipathicity, which influences host-cell toxicity and bacterial selectivity; and **(F)** structural stabilization through cyclization and D-amino acid incorporation. Together, these features influence peptide stability, selectivity, toxicity, and clinical anti-MDR potential.

To understand what sets apart certain (AMPs) that maintain efficacy against multidrug-resistant (MDR) pathogens from others that do not under physiological conditions, it is essential to understand basic *in vitro* minimum inhibitory concentration (MIC) assessments. Conventional MIC measurements alone are insufficient to establish activity under physiological conditions. Candidate prioritization should therefore consider five properties: activity at physiological ionic strength, limited serum inactivation, protease stability, structural constraint through defensin engineering, cyclization or mimetic design, and an appropriate balance between cationicity and hydrophobicity (Luo et al., 2022; Xuan et al., 2023; Salehi et al., 2025; Travé-Asensio et al., 2025). The unrelated engineered AMPs d-WRL and W(Dab)L are included only as comparators illustrating the experimental assessment of salt tolerance, serum stability, and protease resistance (Yadav et al., 2025). Their performance is not considered defensin-specific evidence. Together, these criteria distinguish initial antibacterial potency from physiologically robust and translationally relevant activity (**Figure 4**).

### 5.1 Salt sensitivity: ionic strength as a hidden failure point

Many cationic AMPs act through their electrostatic attraction to the lipids in the bacterial membranes which are negatively charged (**Figure 4A**). Physiological concentrations of ions, particularly NaCl, can reduce peptide–membrane interactions and lead to many cationic AMPs losing considerable amounts of antimicrobial activity (Fu et al., 2023; Xuan et al., 2023). Native defensins may also exhibit reduced antimicrobial activity in hypertonic conditions like airway surface liquid, and blood plasma (Zhao et al., 2024). Addressing salt sensitivity is therefore central to anti-MDR development. The discovery of salt-tolerant and protease-stable cationic AMPs effective against MRSA biofilms illustrates how hydrophobic balance and structural constraints can counteract ionic screening mechanisms (Yadav et al., 2025). Structure-activity relationship (SAR) analyses underscore that amphipathicity and hydrophobic moment rather than net charge alone dictate activity retention in physiological salt conditions.

### 5.2 Serum binding and protein sequestration

The binding of serum proteins like albumin and lipoproteins by cationic AMPs lowers their free concentration and reduces their ability to fight bacteria (**Figure 4B**) (Xuan et al., 2023). Hence, high plasma protein binding is a major translational barrier for use of cationic AMPs as therapeutic agents. Comprehensive AMP analyses demonstrate that heightened hydrophobicity enhances nonspecific serum binding, thereby diminishing the therapeutic index (Salehi et al., 2025). Thus, increasing membrane affinity may also increase nonspecific sequestration by host proteins. In order to reduce serum inactivation while preserving the antibacterial efficacy of defensin-like peptides, peptidomimetic strategies have been used to reduce serum inactivation while preserving antibacterial activity (Luo et al., 2022).

### 5.3 Protease susceptibility and biological stability

Proteolytic degradation in serum, inflamed tissues, or infection sites can shorten the half-life of native therapeutic peptides (**Figure 4C**) (Fu et al., 2023). Disulfide-bridged folds give defensins stability and protection from proteases, while linear analogues of AMP may rapidly degrade due to their linear form. As comparator evidence, the unrelated engineered AMPs d-WRL and W(Dab)L retained anti-MRSA and antibiofilm activity after protease exposure (Yadav et al., 2025). This finding illustrates a useful stability-assessment approach but is not evidence of protease resistance in defensins. The optimization of recombinant host defense peptides provides evidence that design constraints and residue selection dramatically influence peptide degradation kinetics (Travé-Asensio et al., 2025). Cyclic defensins (θ-defensin-like) have been shown to possess greater resistance to proteolysis compared to their linear counterparts (Nagib et al., 2025).

### 5.4 Cyclization and D-amino acid incorporation

Cyclization of the backbone enhances structural stability, making it more resistant to enzymatic degradation (**Figure 4D**). The naturally occurring θ-defensins are examples of how cyclization from head to tail facilitates serum stability and conformational integrity (Nagib et al., 2025). Synthetic defensin-like structures and peptidomimetics use conformational restriction to improve pharmacokinetic properties (Luo et al., 2022). Other AMP design frameworks emphasize the difficulty of proteolytic degradation created by the macrocyclic structure and provide an avenue for selective interactions with bacterial membranes rather than host-cell membranes (Xuan et al., 2023). The substitution of D-amino acids has long been recognized as a way to enhance the potential of AMPs by decreasing their susceptibility to degradation by proteases and increasing their half-lives; however, application of this principle does not occur for all defensins (Salehi et al., 2025).

### 5.5 Why “more cationic” is not always better

Cationicity is crucial for defensin function due to the prevalence of negatively charged phospholipids in bacterial membranes (**Figure 4E**). However, an excessive positive charge does not mean an increased antimicrobial potency (Xuan et al., 2023). Excessive cationicity can increase hemolytic or cytotoxic effects on host cells (Salehi et al., 2025) and improve the binding of nonspecific serum proteins (Xuan et al., 2023). Structure-activity analyses indicate that amphipathic balance, rather than maximal charge, serves as the primary predictor of selective bacterial targeting (Travé-Asensio et al., 2025). Certain defensins also work by binding to specific molecules (like lipid II or ATPase) in ways that depend more on the shape of the molecules than on how strongly they attract each other (Andrés et al., 2024; Jekhmane et al., 2024).

## 6 Mechanisms of action mapped to bacterial envelope biology

Defensins play a crucial role in a wide range of biological processes, including antibacterial and antiviral defense, anticancer activity, and immune regulation. These diverse functions, together with their broad-spectrum antimicrobial activity and unique mechanisms of action, have attracted considerable attention for their therapeutic potential. Consequently, several defensins have been extensively investigated, with some advancing to clinical trials and demonstrating encouraging outcomes (Zhao et al., 2024). More broadly, many AMPs disrupt bacterial membranes or engage multiple cellular targets rather than acting through a single intracellular target. This mechanistic breadth may reduce dependence on any single resistance mutation, but it does not establish a uniformly low probability of resistance. Resistance liability must therefore be evaluated for each peptide–pathogen combination under defined experimental conditions (Xuan et al., 2023). This distinctive mode of action has positioned AMP-based therapeutics as a potential strategy to address antimicrobial resistance while reducing the limitations associated with conventional antibiotic treatment (Zhao et al., 2024).

### 6.1 Human defensins as natural antibacterial peptides

**Figure 5:**
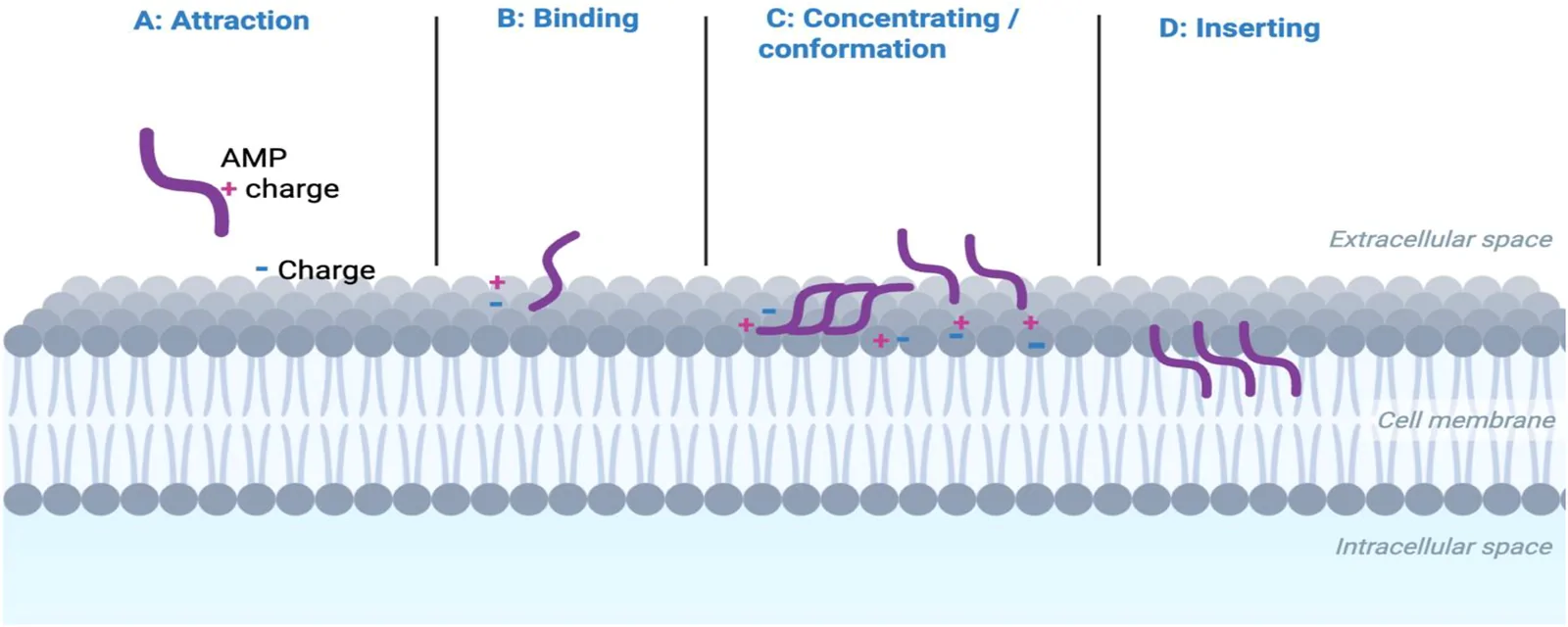
Sequential early events in defensin interaction with the bacterial membrane. Cationic defensins are initially attracted to the negatively charged bacterial surface, followed by membrane binding, local peptide accumulation, membrane perturbation, and insertion into the lipid bilayer.

Human defensins (HDs) are capable of inhibiting bacterial growth or killing bacteria through several antimicrobial mechanisms (Xu and Lu, 2020). Moreover, HDs contribute to decreasing bacterial infection severity by neutralizing toxins released by bacteria. For instance, human α-defensins have been shown to inactivate anthrax lethal toxin, thereby protecting against its lethal effects (Scudiero et al., 2020). Antibacterial mechanisms depend on the defensin subclass and the individual peptide, due to their structural differences. Some α-defensins exhibit greater hydrophobicity and a lower net positive charge than certain β-defensins.;. Bacterial membranes, whether Gram-positive or Gram-negative, contain negatively charged phospholipids that are stabilized by divalent cations such as magnesium and calcium (Nagib et al., 2025). In contrast, membranes of human cells mainly consist of neutral phospholipids. Because defensins carry a positive charge, they are electrostatically attracted to the negatively charged bacterial surface (**Figure 5**), enabling them to bind to the membrane and disrupt the electrostatic interactions that maintain membrane integrity, ultimately resulting in bacterial membrane damage (Scudiero et al., 2020).

Defensin-mediated bacterial killing often begins with electrostatic attraction between cationic peptide residues and negatively charged bacterial surface components, followed by membrane binding, local peptide accumulation, and membrane perturbation (Li et al., 2017; Fu et al., 2023). These effects are commonly interpreted through three simplified membrane-disruption models: barrel-stave, toroidal pore, and carpet-like disruption (Li et al., 2017). In the barrel-stave model, peptides insert approximately perpendicular to the lipid bilayer and oligomerize to form aqueous pores, with hydrophobic peptide regions facing the lipid core and hydrophilic regions lining the pore. In the toroidal pore model, peptide insertion induces membrane curvature, producing pores lined jointly by peptide surfaces and phospholipid head groups (Li et al., 2017). In the carpet model, peptides accumulate on the membrane surface until a threshold concentration is reached, after which the bilayer is destabilized in a detergent-like manner rather than through stable pore formation (Li et al., 2017). However, these models should be presented cautiously for defensins, because individual defensins may use mixed, lipid-specific, or context-dependent mechanisms rather than a single universal pathway (Fu et al., 2023). Structural evidence for the plant defensin NaD1 supports this view by showing a carpet-like defensin–phospholipid disruption complex (Järvå et al., 2018). Accordingly, barrel-stave, toroidal pore, and carpet models are best used as conceptual frameworks, while defensin activity should be interpreted in relation to peptide structure, membrane lipid composition, and local peptide concentration.

**Figure 6:**
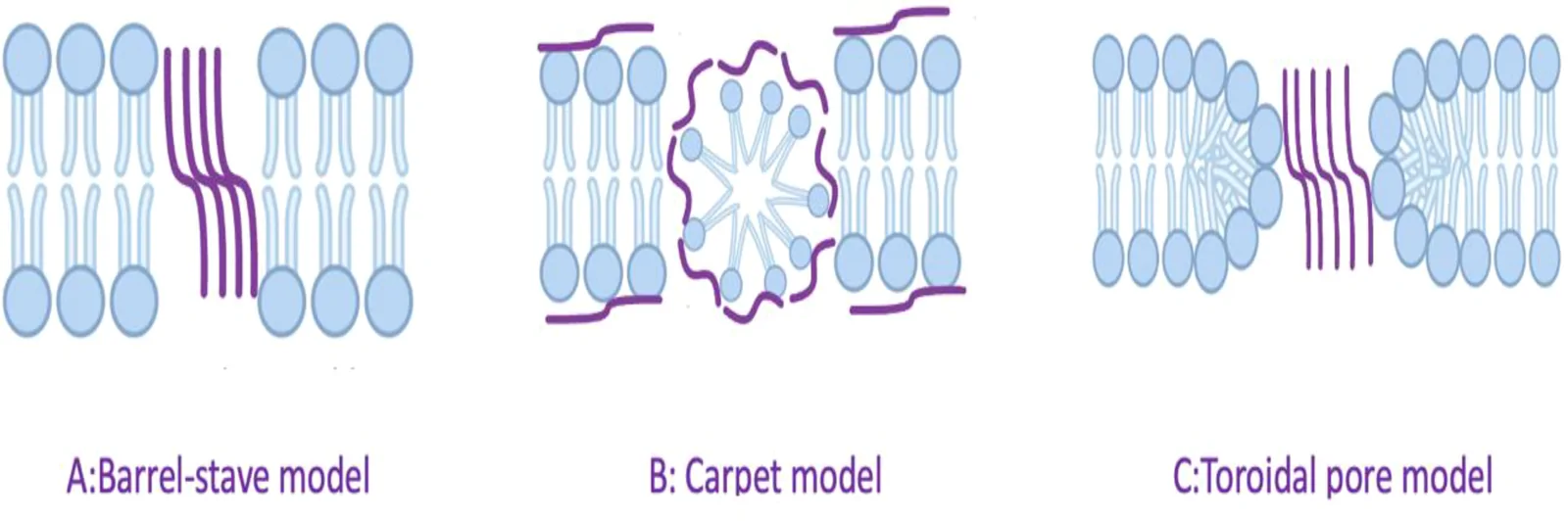
Conceptual membrane-disruption models relevant to defensin activity. Following membrane engagement, defensins may induce **(A)** barrel-stave pores, **(B)** carpet-like bilayer disruption, or **(C)** toroidal pores. These general AMP models provide mechanistic frameworks and should not be interpreted as universal or experimentally confirmed pathways for all defensins.

For mechanistic comparison, Peptide 4, an unrelated microbiome-derived AMP, illustrates concentration-dependent membrane disruption in MRSA. Following metagenomic identification and chemical synthesis, it caused membrane depression and leakage at 1× MIC and cellular collapse and lysis at 2× MIC, consistent with membrane permeabilization and fluidization (Shen et al., 2025). This comparator evidence is not interpreted as demonstrating an equivalent mechanism in defensins. Following the initial membrane-engagement sequence illustrated in **Figure 5**, defensin-induced membrane disruption may proceed through several conceptual pathways, summarized in **Figure 6**.

### 6.2 Fungal defensin plectasin as a lipid II–directed antibacterial peptide

**Figure 7:**
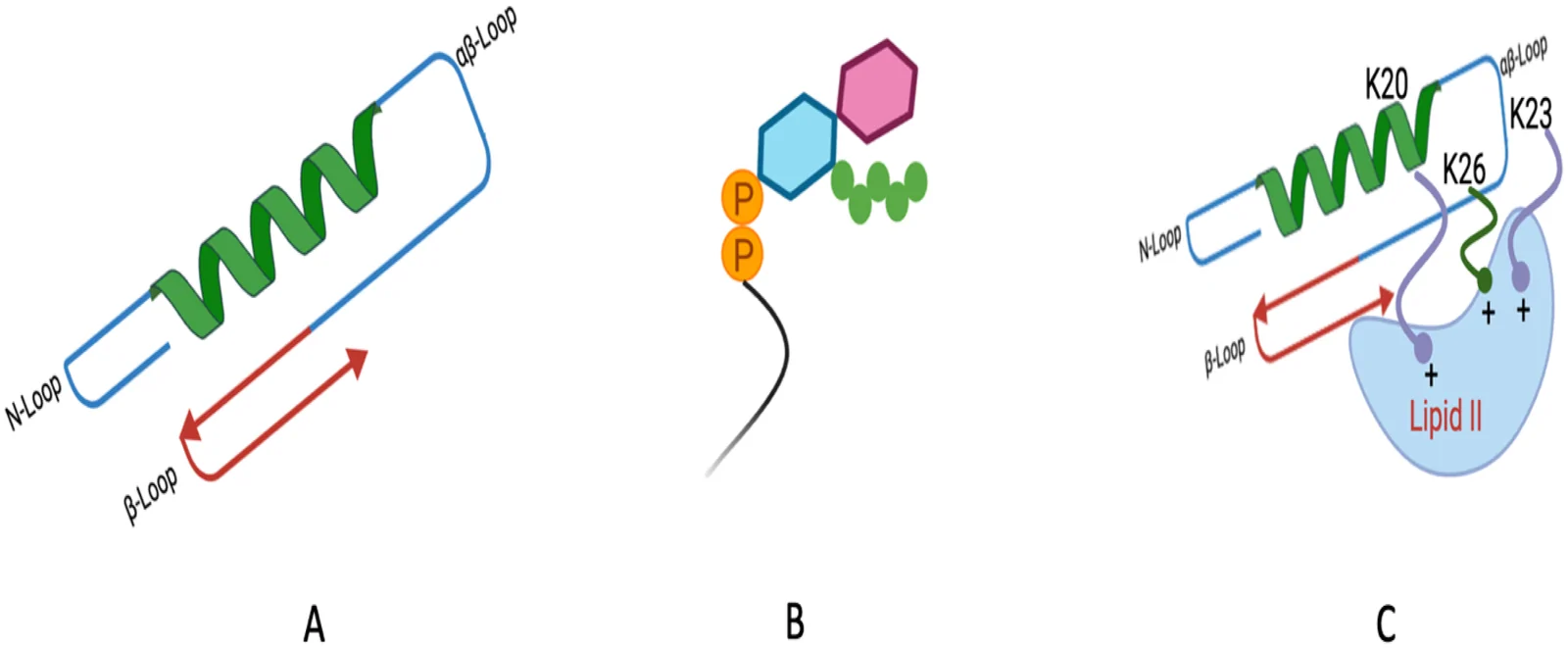
Structural basis of plectasin binding to lipid II. **(A)** Plectasin interaction with the peptidoglycan precursor lipid II in the plasma membrane, **(B)** Lipid II complex, **(C)** The Plectasin’s αβ-loop with the free hydrogen bonding cationic residues (K20, K23, K26) directly involved in the coordination of anionic lipid II head-group.

The identification of Plectasin, a host defense peptide isolated from the fungus *Pseudoplectania nigrella*, generated significant scientific interest (Melcrová et al., 2023). The peptide and its variants exhibit potent effect against pathogens such as *Mycobacterium tuberculosis*, methicillin-resistant *Staphylococcus aureus* and *Streptococcus pneumoniae* (van Beekveld et al., 2022). Plectasin (**Figure 7A**) displays its antibacterial activity by interacting with the peptidoglycan precursor lipid II in the plasma membrane and disrupting the cell wall biosynthesis. Lipid II (**Figure 7B**) is a complex C55-polyisoprenoid-based lipid with a conserved pyrophosphate (PPi) group, a headgroup composed of the sugars MurNAc and GlcNAc and a pentapeptide.

Plectasin’s αβ-loop plays a crucial role in lipid II binding and antimicrobial activity since the αβ-loop region contains several cationic residues capable of forming hydrogen-bonding and electrostatic interactions (K20, K23, K26) directly involved in the coordination of anionic lipid II head-group (**Figure 7C**) (Jekhmane et al., 2024). In a recent study, Jekhmane et al (Jekhmane et al., 2024), explained how the interaction between plectasin with the essential cell wall precursor lipid II within bacterial membranes occurs. Plectasin utilizes the hydrophobic amino acids located in the N-loop and αβ-loop regions to embed itself within the bacterial membrane surface. Once associated, Plectasin immediately attaches to pyrophosphate group, the MurNAc sugar, and the pentapeptide chain, the conserved structural elements of lipid II. At this stage, evidence from ssNMR, ITC, and fluorescence spectroscopy indicates that the extended αβ-loop folds into a stable structure which is crucial for lipid II recognition and binding. This structure likely stabilizes interactions between positively charged lysine side chains of Plectasin and the negatively charged groups, such as the pyrophosphate moiety or the γ-glutamate, of the lipid II pentapeptide (Jekhmane et al., 2024). Plectasin is a fungal defensin whose antibacterial activity is primarily mediated through direct binding to the bacterial cell-wall precursor lipid II, thereby interfering with peptidoglycan biosynthesis (Schneider et al., 2010). More recent structural and biophysical evidence further showed that Ca²^+^ enhances plectasin activity by stabilizing key lipid II-binding regions and promoting a calcium-sensitive supramolecular mechanism.

Additionally, Ca²^+^ ions enhance the formation of dense, carpet-like plectasin–lipid II assemblies on the membrane surface. In addition, oligomerization is an important component of the peptide’s antimicrobial mechanism, which occurs at concentrations near minimum inhibitory concentration (MIC). These rigid supramolecular structures appear to be effectively irreversible and function as a sink that gathers lipid II molecules into a network where they become trapped. Such assemblies disrupt the normal fluidity of the bacterial membrane, which may contribute to plectasin’s antimicrobial activity. The oligomerization process depends strongly on conserved histidine residues located on opposite sides of the plectasin molecule. Shukla et al. showed that replacing these residues with alanine significantly weakens lipid II binding affinity, indicating that lipid II recognition and oligomer formation are closely linked. A similar relationship between oligomerization and antimicrobial function has been observed for other lipid II-targeting antibiotics, such as teixobactin (Shukla et al., 2022). Importantly, plectasin does not attach to soluble pyrophosphate or pyrophosphate-containing nucleosides existing in the environment. Instead, strong interaction with the pyrophosphate group occurs only when supramolecular assemblies form on the membrane surface, which significantly reduces the rate of dissociation and increases the residence time of plectasin at its target. This mechanism explains how plectasin selectively recognizes sugar-pyrophosphate motifs of cell wall precursors such as lipid I and lipid II while remaining non-toxic to mammalian cells (Jekhmane et al., 2024).

### 6.3 Peptidomimetics as intracellular-targeted antibacterial peptides

Earlier work mainly linked the antibacterial activity of peptidomimetic antibiotics to membrane disruption. However, Luo et al. later showed that a human defensin-inspired peptidomimetic, compound 10, may also act through an intracellular mechanism involving the bacterial 70S ribosome. In *E. coli*, compound 10 showed strong interaction with the 70S ribosome, with ribosome binding reported to be approximately 30-fold stronger than its interaction with lipopolysaccharide and with a low dissociation constant. Functional assays further indicated that this interaction interfered with ribosome-mediated protein synthesis. Therefore, for compound 10, antibacterial activity appears to involve a dual mechanism: disruption of the Gram-negative bacterial envelope and inhibition of intracellular protein synthesis. This dual targeting may contribute to its potent bactericidal activity, although this mechanism should not be generalized to all peptidomimetic antibiotics without compound-specific evidence (Luo et al., 2022).

### 6.4 Plant and insect defensins as natural antibacterial agents

Plant defensins exhibit antibacterial activity against strains of *Pseudomonas aeruginosa*. A defensin from *Medicago truncatula* named MtDef4 induced dose-dependent gene expression of the amino arabinose modification of bacterial Lipopolysaccharides (LPS) and surface polycation spermidine production operons. The antibacterial effect of MtDef4 by damaging bacterial outer membranes was visually verified via fluorescent microscopy. However, MtDef5, another *M. truncatula*’s defensin, failed to trigger such gene expression and caused less outer-membrane damage (Sathoff et al., 2020). Interestingly, the larva *Hermetia illucens*, following infection with *Salmonella enterica* serovar Typhimurium, expressed potent AMP namely Jg7197.t1, Jg7902.t1 and Jg7904.t1 into the hemolymph. Therefore, in this study it was revealed that the 3 AMPs shared primary sequences with proteins that contain a Kunitz-binding domain; characterized for inhibitory action against proteases, and antimicrobial activities. Moreover, this study showed that cloned and purified putative AMPs had antimicrobial activity against a range of Gram-negative and Gram-positive bacteria, including the serious pathogen *Pseudomonas aeruginosa* (Fahmy et al., 2024).

### 6.5 Human defensin 6: entrapment-driven mechanism for drug design

Six α-defensins have been identified in humans, including human neutrophil peptides 1- 4 (HNP1-4), the enteric defensins human defensin 5 (HD5) and human defensin 6 (HD6). These peptides share a conserved disulfide connectivity pattern formed during oxidative folding. HNP1–4 is primarily stored in the azurophilic granules of neutrophils and are also present in monocytes and natural killer cells. In contrast, HD5 and HD6 are highly concentrated within the secretory granules of Paneth cells located at the base of the crypts of Lieberkühn in the small intestine. Therefore, Paneth cells contribute to intestinal immunity by secreting a variety of antimicrobial peptides and proteins, including HD5 and HD6, which protect the intestinal epithelium from infection and colonization by pathogenic or opportunistic microorganisms (Lei et al., 2025). Although all six human α-defensins share a conserved disulfide-stabilized fold, their sequences and functions differ markedly. HD5 (32 aa) forms dimers/tetramers and shows broad-spectrum antibacterial activity, whereas HD6 self-assembles into higher-order fibrillar “nanonets” that trap bacteria and prevent epithelial invasion. Structural data indicate that hydrophobic residues of HD6 (e.g., F2, V22, M23, I25, F29, L32) cluster to form inter-monomer pockets that drive this assembly. This non-lytic entrapment mechanism highlights how hydrophobic patterning within the α-defensin scaffold modulates function and offers a strategy to limit resistance by immobilizing, rather than killing, pathogens (Gera et al., 2022).

## 7 Evidence against priority MDR pathogens

### 7.1 Methicillin-resistant Staphylococcus aureus

Methicillin-resistant *Staphylococcus aureus* (MRSA) exerts a challenging health problem because of the mechanisms of resistant toward penicillin antibiotics. It is the leading cause of several life-threatening conditions like endocarditis, septicemia, joints, bone, skin and soft tissue infections in clinical and community settings and is associated with substantial morbidity and mortality (Nagib et al., 2025). Several mechanisms of resistance were attributed to MRSA such as decreased affinity for β-lactam antibiotics due to the presence of the *mecA* gene, reduced intracellular concentration of antibiotic due to efflux pump and the formation of biofilm to act as a defense barrier against antibiotics (Hassanzadeh et al., 2020). Recently, defensin-based therapies have emerged as a novel and innovative option for the treatment of MRSA infections. hBD-1, hBD-2, and hBD-3 contribute in the immune response against *S*. *aureus*, including MRSA. Out of the three previously mentioned HDs, HBD3 showed the strongest antibacterial activity against 44 clinical isolates of *S*. *aureus*, 22 of which were MRSA strains. However, approximately half of the MRSA strains, exhibited more than 20% survival with HBD3, suggesting that there are still some resistance mechanisms against the activity of HDs. Another theorized method related to mechanism of resistance mechanism to HDs is the production of Staphylokinase (SAK), a serine protease-like molecule. SAK was found to neutralize the bactericidal effects of human neutrophil peptides (HNPs). Cell-free SAK triggers the secretion of HNPs, after release, SAK immediately binds to the HNPs causing complex formation. This mechanism effectively neutralizes HDs, preventing them from binding to the bacterial surface (Nagib et al., 2025). On the other hand, nanomedicine is being investigated as a strategy to enhance antimicrobial therapy against MRSA infections. By combining nanoparticles with antimicrobial peptides (AMPs), nanomedicine may improve peptide stability, delivery, and target specificity. Metallic nanoparticles, such as silver and zinc oxide, not only act as carriers but also possess intrinsic antimicrobial activity that synergizes with AMPs. Gold nanoparticles further enhance AMP potency while decreasing toxicity and improving serum stability. Magnetic nanoparticles and polymeric nanoparticles offer sustained-release properties with reduced toxicity, making them ideal for effective drug delivery systems (Yin et al., 2020). Nevertheless, meaningful comparison of AMP performance across studies remains challenging due to differences in peptide design, susceptibility testing methods, and the diversity of MRSA strains evaluated. Since MRSA frequently causes chronic wound, skin and soft tissue, bloodstream, and implant-associated infections, where biofilm formation plays a central role in persistence and antibiotic tolerance, assessing AMP efficacy against both planktonic and biofilm-associated bacteria is essential (Chen et al., 2023). In addition, physiological factors such as serum proteins and proteolytic enzymes may reduce peptide stability and antimicrobial activity, emphasizing the importance of evaluating AMPs under clinically relevant conditions. Comprehensive assessment of hemolytic activity, cytotoxicity, and pharmacokinetic properties is also required to establish an adequate therapeutic index. Although numerous AMPs have demonstrated potent *in vitro* activity against MRSA, further optimization of peptide stability, delivery strategies, and *in vivo* efficacy is necessary to facilitate their translation into effective therapeutic agents (Zhang et al., 2024).

### 7.2 Carbapenem-resistant *Acinetobacter baumannii*

A total of forty-six peptides with activity against *Acinetobacter* were identified and organized into ten categories: cathelicidins, defensins, frog-derived AMPs, melittin, cecropins, mastoparan, histatins, dermcidins, tachyplesins, in addition to computationally designed peptides. Based on reported minimum inhibitory concentrations (MICs), the defensin group, particularly α- and β-defensins, exhibited the strongest reported activity against both antibiotic-sensitive and resistant *A. baumannii* strains, their antimicrobial mechanisms generally fall into two categories: those targeting bacterial membranes and those acting on intracellular components (Neshani et al., 2020). HNPs and HD5 (α-Defensins) contain three pairs of disulfide bonds and have demonstrated strong antibacterial activity against *Acinetobacter* species. Accordingly, HNP-1 inhibits the growth of the standard strain *A. baumannii* ATCC 19606 at 50 μg/mL, whereas a colistin-resistant mutant of the same strain is significantly more sensitive, with an MIC of 3.25 μg/mL. On the other hand, HD5 showed more modest activity against *A. baumannii*, with an MIC of 320 μg/mL. However, a modified analog HD5d5, showed enhanced bactericidal activity where the MIC value dropped significantly to 40 μg/mL. The reported antibacterial effect of defensins involves disrupting bacterial membranes, accumulating inside the cytoplasm, and reducing superoxide dismutase and catalase activity (Neshani et al., 2020). On the other hand, members such as HBD-2 and hBD-3 confirmed that they exhibit activity against *Acinetobacter* species with potent antibacterial activity against multidrug-resistant (MDR) clinical isolates than non-MDR strains. Although, the antibacterial activity of hBD-3 diminishes in the presence of human serum, hBD-3 can kill all clinical *A. baumannii* isolates, including MDR and non-MDR strains, at 4 μg/mL within 1.5 hours in serum-free conditions. Additionally, its wound-healing properties make it a promising candidate for treating *A. baumannii*-infected wounds particularly in patients with chronic conditions, including diabetes (Rasooli et al., 2020). However, comparing AMPs**’** reported efficacy requires careful interpretation due to variations in susceptibility testing protocols, assay conditions, and the use of reference strains versus genetically diverse clinical CRAB isolates (Zhang et al., 2024). In addition, *A. baumannii* is characterized by exceptional environmental persistence, desiccation tolerance, and robust biofilm-forming capacity, all of which contribute to enhanced antimicrobial tolerance and treatment failure. Consequently, evaluating AMP activity against mature biofilms and under physiologically relevant conditions, including the presence of serum and physiological salt concentrations, is essential for predicting their therapeutic performance (Chen et al., 2023). Equally important is the assessment of peptide stability, hemolytic activity, and cytotoxicity to ensure a favorable therapeutic index before clinical application. Although numerous AMPs have demonstrated potent *in vitro* activity against CRAB, their successful clinical translation will ultimately depend on improved stability, reduced host toxicity, scalable production, and validation of efficacy in animal infection models (Zhang et al., 2024).

### 7.3 Multidrug-resistant *Pseudomonas aeruginosa*

*Pseudomonas aeruginosa* is a prominent opportunistic Gram-negative pathogen and a major cause of hospital-acquired infections, including pneumonia, bacteremia, and urinary tract infections. Immunocompromised individuals, particularly those with metabolic disorders, hematological diseases, or malignancies, are at increased risk. Excessive and inappropriate antibiotic use has accelerated resistance development, especially to carbapenems, resulting in multidrug-resistant strains and serious therapeutic challenges (Chen et al., 2023). Several AMPs have demonstrated strong anti-*P. aeruginosa* activity of which Plant defensins, these peptides demonstrate broad antimicrobial activity, inhibiting the growth of numerous pathogens such as filamentous fungi and *P. aeruginosa*. Several peptides isolated from spinach leaves have shown antimicrobial properties. In addition, Defensin-d2 was able to inhibit biofilm formation by *P. aeruginosa* in a dose-dependent manner, where the treatment with 3.75 μg/mL of Defensin-d2 for 24 hours reduced the biofilm biomass by more than 70% (Gbala et al., 2022). The proposed mechanism resembles that of actifensin, involving enhanced production of reactive oxygen species (ROS) and damage to the bacterial cell membrane. On the other hand, the plant *Medicago truncatula* also produces several peptides with antimicrobial activity against human pathogens. Sathoff et al. reported that core MtDef4 and MtDef5, derived from *Medicago truncatula* and having molecular weights of 1.98–2.04 kDa, inhibited the growth of *P. aeruginosa* with IC_50_ values of 1.7–4.2 μM and 8.5–14.6 μM, respectively (Sathoff et al., 2020). Furthermore, core MtDef4 was found to induce the expression of genes associated with aminoarabinose modification of LPS on the bacterial surface, resulting in the damage of the outer membrane of *P. aeruginosa*. In contrast, MtDef5 appears to disrupt DNA synthesis and transcription processes (Chen et al., 2023). However, direct comparisons of AMP efficacy remain challenging because reported antimicrobial potency is influenced by substantial methodological variability. Minimum inhibitory concentrations (MICs) differ depending on assay conditions, including growth medium composition, inoculum size, incubation time, pH, ionic strength, and the presence of divalent cations, all of which can alter peptide activity (Mookherjee et al., 2020). Another important consideration is the loss of antimicrobial activity under physiological conditions, as many AMPs exhibit reduced efficacy in the presence of serum proteins, physiological salt concentrations, or host proteases, which can impair peptide stability and bioavailability (Chen et al., 2023). Equally critical is the assessment of host toxicity, including hemolytic activity and cytotoxicity toward mammalian cells, as an ideal AMP should display a high therapeutic index while maintaining potent antibacterial activity. Because *P. aeruginosa* commonly establishes biofilm-associated infections, evaluation against mature biofilms rather than planktonic bacteria alone provides a more clinically relevant measure of efficacy (Scudiero et al., 2020). Finally, the translational potential of AMPs depends not only on *in vitro* antimicrobial potency but also on favorable pharmacokinetic properties, resistance to enzymatic degradation, low immunogenicity, scalable manufacturing, and demonstrated efficacy in relevant animal infection models. Therefore, comprehensive evaluation using standardized susceptibility testing, diverse clinical isolates, physiological assay conditions, biofilm models, toxicity assessments, and *in vivo* validation is essential for accurately determining the therapeutic promise of AMPs against *P. aeruginosa* infections (Mookherjee et al., 2020).

### 7.4 Carbapenem-resistant Enterobacteriaceae

Evidence supporting defensins against carbapenem-resistant Enterobacterales remains limited but includes native defensins and defensin-inspired candidates. hBD-3, a native human β-defensin, showed activity against 23 carbapenem-resistant *Klebsiella pneumoniae* and 17 carbapenem-resistant *Klebsiella aerogenes* isolates, with median MICs of 4 mg/L for both species. Its combination with epinecidin-1, an unrelated fish-derived AMP, produced isolate-dependent rather than uniform synergy. In a murine sepsis model, 120-h survival reached 63.3% with hBD-3 and 83.7% with hBD-3/meropenem, compared with 0% for untreated and meropenem-treated animals. However, the animal experiment used a single *K. pneumoniae* isolate and a single-dose regimen, limiting generalizability (Bolatchiev, 2022).

Further translational evidence comes from MTD12813, a minimized θ-defensin-inspired cyclic peptide rather than a native defensin. It was evaluated against carbapenem-resistant *K. pneumoniae* and *E. coli* expressing KPC-2 or NDM-1. A single dose improved survival in murine septicemia by enhancing phagocytosis, bacterial clearance, and immune regulation. Notably, MTD12813 lacked direct antibacterial activity in mouse serum and peritoneal fluid, indicating a predominantly host-directed mechanism. It also showed biological-matrix stability, bacterial-protease resistance, and tolerability at doses exceeding the effective dose by more than 100-fold (Schaal et al., 2021). These findings should therefore be interpreted as evidence for a defensin-inspired candidate, not for native human defensins as a class.

Complementary studies indicate that defensin activity against *K. pneumoniae* may also involve antibiofilm and immunomodulatory effects. hBD-3 reduced biofilm formation across three *K. pneumoniae* strains, although planktonic growth was only weakly affected under the tested conditions (Hanstein et al., 2025). Similarly, prophylactic intranasal administration of bovine neutrophil β-defensin-5 reduced pulmonary and splenic bacterial burdens and limited lung injury in mice challenged with MDR *K. pneumoniae*, despite lacking direct *in vitro* bactericidal activity (Zhu et al., 2024). Because these studies did not specifically select carbapenem-resistant isolates, they provide mechanistic context rather than direct CRE efficacy evidence.

Collectively, the evidence identifies two potential development routes: direct antibacterial activity and antibiotic potentiation, exemplified by hBD-3, and host-directed immunomodulation, exemplified by MTD12813 and bovine β-defensin-5. Nevertheless, efficacy remains candidate-, strain-, and assay-dependent. Future studies should include diverse carbapenemase genotypes, capsular types, physiological matrices, pharmacokinetic and toxicity assessments, resistance-selection experiments, and therapeutic, and not solely prophylactic animal models.

### 7.5 Neisseria gonorrhoeae

*Neisseria gonorrhoeae*, the causative agent of gonorrhea, is a major public-health concern because resistance has emerged to nearly every antimicrobial class used for treatment. Brilacidin, a synthetic defensin mimetic rather than a native defensin or defensin-derived peptide, showed potent activity against multidrug-resistant clinical isolates of *N. gonorrhoeae*, including ceftriaxone-resistant strains. Its antibacterial activity was associated with increased propidium iodide uptake and extracellular ATP release, both of which indicate loss of cytoplasmic-membrane integrity. Separately, the MtrCDE efflux pump contributes to gonococcal survival during exposure to human neutrophils and selected neutrophil-derived AMPs (Handing et al., 2018). Whether MtrCDE alters brilacidin susceptibility, or whether brilacidin-mediated membrane injury compromises pump function, has not been experimentally determined.Because brilacidin disrupts membrane integrity and dissipates the proton gradient, it may impair MtrCDE function, although this proposed interaction requires direct experimental confirmation (Abdelsattar et al., 2025). Across 22 drug-resistant strains, brilacidin produced MICs of 1–8 μg/mL, with MIC_50_ and MIC₉_0_ values of 4 and 8 μg/mL, respectively. At 4× MIC, it eradicated the ceftriaxone-resistant WHO-X strain within 2 h, whereas azithromycin required 6 h (Abdelsattar et al., 2025). The mechanism of action of drugs with rapid bactericidal activity, particularly antimicrobial peptides, is often mediated by disrupting the bacterial membrane. Brilacidin-induced propidium iodide uptake and extracellular ATP leakage in *N. gonorrhoeae* support cytoplasmic-membrane permeabilization (Abdelsattar et al., 2025). Independent bacterial studies have similarly demonstrated brilacidin-mediated membrane depolarization, while recent evidence in *Cryptococcus neoformans* showed disruption of membrane organization and increased permeability (Mensa et al., 2014; Diehl et al., 2024). The latter provides compound-specific mechanistic support but should not be considered direct confirmation of the gonococcal mechanism. MtrCDE is a proton-motive-force-dependent RND efflux system that exports antibiotics and host-derived antimicrobials. Peptides designed specifically to inhibit its components increased gonococcal susceptibility to erythromycin, azithromycin, ciprofloxacin, and ceftriaxone by 2- to 64-fold (Evert et al., 2022). Nevertheless, controlled infection studies indicate that the contribution of MtrCDE to *in vivo* fitness is strain- and model-dependent (Waltmann et al., 2024). No study has demonstrated direct inhibition of MtrCDE by brilacidin; therefore, pump impairment following brilacidin-induced membrane damage remains a mechanistic hypothesis.The principal pathogen-specific activity data discussed in Sections 7.1–7.5 are consolidated in **Table 3** to facilitate comparison of peptide potency, antimicrobial mechanism and supporting evidence.

**Table 3:** Pathogen-specific activity and mechanisms of defensins defensin-derived candidates, defensin mimetics, and comparator AMPs against drug-resistant bacteria.

Target pathogen	Candidate compound(s) and classification	Reported activity	Proposed or supported mechanism	Reference
MRSA	hBD-3 — native human β-defensin	Active against clinical *S. aureus* isolates, including MRSA; approximately half of the MRSA isolates retained >20% survival	Membrane interaction; susceptibility influenced by the membrane-modifying protein FmtC	Escobar-Salom et al. (2024)
MRSA	Peptide 4 — unrelated microbiome-derived AMP comparator	MIC and time-kill activity reported; dose-dependent membrane damage at 1× and 2× MIC	Membrane permeabilization, fluidization, leakage and eventual cellular lysis	Shen et al. (2025)
Carbapenem-resistant *A. baumannii*	HNP-1 — native human α-defensin	MIC: 50 μg/mL against ATCC 19606 and 3.25 μg/mL against a colistin-resistant mutant	Membrane disruption, cytoplasmic accumulation and impairment of oxidative-stress defence	Neshani et al. (2020)
Carbapenem-resistant *A. baumannii*	HD5 and HD5d5 — native human α-defensin and defensin-derived analogue	MIC: 320 μg/mL for HD5 and 40 μg/mL for HD5d5	Membrane disruption and intracellular effects; activity enhanced by peptide modification	Neshani et al. (2020)
MDR *A. baumannii*	hBD-3 — native human β-defensin	Killing at 4 μg/mL within 1.5 h under serum-free conditions; activity decreased in human serum	Rapid membrane-directed bactericidal activity	Rasooli et al. (2020)
MDR *P. aeruginosa*	Defensin-d2 — recombinant plant defensin	3.75 μg/mL reduced biofilm biomass by >70% after 24 h	Membrane permeabilization and increased reactive oxygen species production	Gbala et al. (2022)
*P. aeruginosa*	Core MtDef4 and core MtDef5 — plant defensin-derived peptides	IC_50_: 1.7–4.2 μM for MtDef4 and 8.5–14.6 μM for MtDef5	MtDef4: outer-membrane damage and induction of LPS-remodelling responses; MtDef5: proposed interference with DNA synthesis and transcription	Sathoff et al. (2020)
Carbapenem-resistant *K. pneumoniae* and *E. coli*	hBD-3 with epinecidin-1 or antibiotics — native defensin with an unrelated fish-derived AMP comparator or conventional antibiotics	Synergy reported with epinecidin-1 against carbapenem-resistant *K. pneumoniae* and with amikacin against resistant *E. coli*; MIC/FICI testing performed	Membrane permeabilization combined with antimicrobial and immunomodulatory effects	Bolatchiev (2022)
MDR *N. gonorrhoeae*	Brilacidin — synthetic defensin mimetic	MIC_50_/MIC₉_0_ and rapid time-kill activity demonstrated against 22 MDR isolates, including ceftriaxone-resistant strains	Cytoplasmic-membrane disruption, propidium iodide uptake and ATP leakage; impairment of MtrCDE is proposed but not directly established	Abdelsattar et al. (2025)

Candidates are classified by their relationship to defensins. Unrelated AMPs are included only as comparators, and their findings should not be interpreted as defensin-specific evidence. Reported values should be compared cautiously because experimental conditions and susceptibility methods differed among studies.

### 7.6 From antibacterial activity to translational readiness: a study-level appraisal

Recent defensin research has progressed unevenly across the translational pathway. Molecular studies have resolved target recognition, supramolecular assembly, and membrane interactions in considerable detail (Jekhmane et al., 2024; Luo et al., 2022). By contrast, physiological stability, host safety, resistance selection, pharmacological exposure, and animal efficacy have often been evaluated separately rather than integrated within the development program for the same candidate, as illustrated by the complementary but incomplete evidence packages reported for recent anti-MRSA peptides (Shen et al., 2025; Yadav et al., 2025). Consequently, compelling evidence in one domain should not be interpreted as evidence of overall therapeutic readiness. Precise definition of target engagement strengthens mechanistic plausibility but does not establish whether an active peptide concentration can be achieved and maintained at the infection site without unacceptable host toxicity. Conversely, efficacy in an animal model remains dependent on the particular scaffold, formulation, dose, pathogen, and infection context and therefore cannot be extrapolated across the structurally and functionally diverse defensin family. Credible translational potential ultimately requires convergence of mechanistic, microbiological, stability, safety, resistance, pharmacokinetic, and *in vivo* evidence within the same candidate, rather than the accumulation of favorable findings from unrelated peptides or experimental systems (Mookherjee et al., 2020; Zheng et al., 2025). The extent to which representative studies achieve this convergence is summarized in **Table 1**.

Three cross-study patterns were evident. First, mechanism-rich and isolate-rich studies generally established target engagement, antibacterial spectrum, or activity against MDR organisms but frequently left physiological performance and host exposure unresolved, as illustrated by the mechanistic investigation of plectasin and the evaluation of human defensins against MDR Gram-negative clinical isolates (Jekhmane et al., 2024; Escobar-Salom et al., 2024). Second, some engineered candidates combined salt tolerance, protease stability, host-safety assessment, and resistance-selection testing without progressing to an infection model (Yadav et al., 2025). Conversely, other candidates demonstrated efficacy in animal infection models without equally direct evaluation of serum activity, proteolytic stability, or exposure–response relationships (Bolatchiev, 2022; Shen et al., 2025). These evidence packages represent different stages of development and should not be treated as equivalent demonstrations of therapeutic feasibility.

Third, serum effects constituted the most consistent evidence gap: none of the representative studies summarized in **Table 1** directly quantified retained antibacterial activity in serum. This endpoint cannot be inferred from low hemolysis, the use of serum-containing cell-culture medium, or pharmacokinetic persistence because these measurements address different biological questions. In particular, binding to albumin and other serum components may decrease the unbound, microbiologically available peptide concentration despite apparently adequate total exposure (Xuan et al., 2023; Zheng et al., 2025). Likewise, animal survival alone does not define a therapeutic window without corresponding pharmacokinetic exposure, bacterial-burden reduction, comparator treatment, and safety data, although studies integrating pharmacokinetic stability with infection-model efficacy provide a stronger preclinical foundation (Luo et al., 2022). Resistance liability also cannot be inferred from rapid killing, membrane disruption, or engagement of multiple targets; it requires direct serial-exposure or resistance-frequency experiments conducted under defined conditions (Gbala et al., 2022; Yadav et al., 2025).

Accordingly, NR was treated as an evidentiary limitation rather than as an unfavourable result. Candidates supported predominantly by MIC, mechanistic, or antibiofilm findings were interpreted as antibacterial leads. Candidates additionally supported by physiological-stability and host-safety data were considered optimized leads, whereas stronger claims of preclinical translational potential required relevant *in vivo* efficacy together with pharmacological and resistance-liability assessment. No representative study in **Table 1** satisfied the complete evidence chain. This calibrated interpretation prevents promising antibacterial activity from being presented prematurely as demonstrated therapeutic feasibility.

## 8 Anti-biofilm activity and device/wound contexts

Biofilm-related infections are one of the most difficult types of multidrug resistance (MDR) to treat. In biofilms, bacteria show less metabolic activity, changes in gene expression, protection by the extracellular polymeric substance (EPS) matrix, and substantially increased tolerance to antibiotics. Antibiofilm activity includes multiple outcomes that are unique and mechanistically different from each other like preventing the initial adhesion of bacteria, inhibiting biofilm formation, disrupting the extracellular matrix, killing bacteria within an already formed biofilm, eliminating mature biofilms, and enhancing the activity of antibiotics. They should not be treated as equivalent outcomes. Additionally, defensins and defensin-like antimicrobial peptides have been shown to exhibit activity in many of these outcomes depending on the particular peptide used and the type of *in vitro* model system. In this case, defensins and defensin-like AMPs have unique benefits because they quickly target membranes, modulate the immune system, and can be used to immobilize surfaces or deliver drugs topically (Xuan et al., 2023; Salehi et al., 2025).

### 8.1 Biofilm penetration: overcoming the extracellular matrix barrier

Infections in diabetic patients with chronic non-healing wounds are difficult to be treated with antibiotics, especially when caused by MDR pathogen with capability to produce biofilm, and these pathogens are root cause death in many cases. On the other hand, most antibacterial agents have effect against planktonic growing bacteria with low to null action against biofilm. Recently, AMPs have shown promising effect toward MDR bacterial infections as a novel antibiofilm therapeutics. Antibiofilm performance should be evaluated using distinct endpoints, including the minimum biofilm inhibitory concentration for prevention of biofilm formation and the minimum biofilm eradication concentration for activity against established biofilms. These endpoints should not be inferred from planktonic MIC values. The hBD-3 showed significant bactericidal effect towards MRSA, MDR methicillin-resistant *Staphylococcus epidermidis* (MRSE), MDR *P. aeruginosa* and MDR *Acinetobacter baumannii*. Moreover, it was shown that defensins provoke osmotic bacterial lysis by binding to bacterial membranes and creating pores (Scudiero et al., 2020). RNA polymerase, DNA gyrase, cell wall, bacterial membrane and protein synthesis inhibition are the main mechanisms behind the synergy of defensins with antibiotics. On the other hand, defensins can disrupt membrane integrity and facilitate antibiotic penetration, thereby enhancing access to bacterial targets. When studied in terms of anti-biofilm action, results also found a synergistic effect of defensins in combination with antibiotics toward biofilm-forming MDR-MRSA and MDR-*P. aeruginosa* especially by degradation of biofilm matrix, and downregulation of genes responsible for biofilm formation, such as the biofilm-associated genes *icaA* and *dltB* genes in *S. aureus* and *LasR* in *P. aeruginosa* respectively. Accordingly, reducing the concentration of used antibiotics in synergism with AMP can decrease the side effects of drug, especially in patients with chronic diseases and kidney failure (Di Somma et al., 2020). The biofilm extracellular matrix, which is made up of polysaccharides, extracellular DNA, and proteins, stops antibiotics from spreading by acting as a physical and electrostatic barrier.

**Figure 8:**
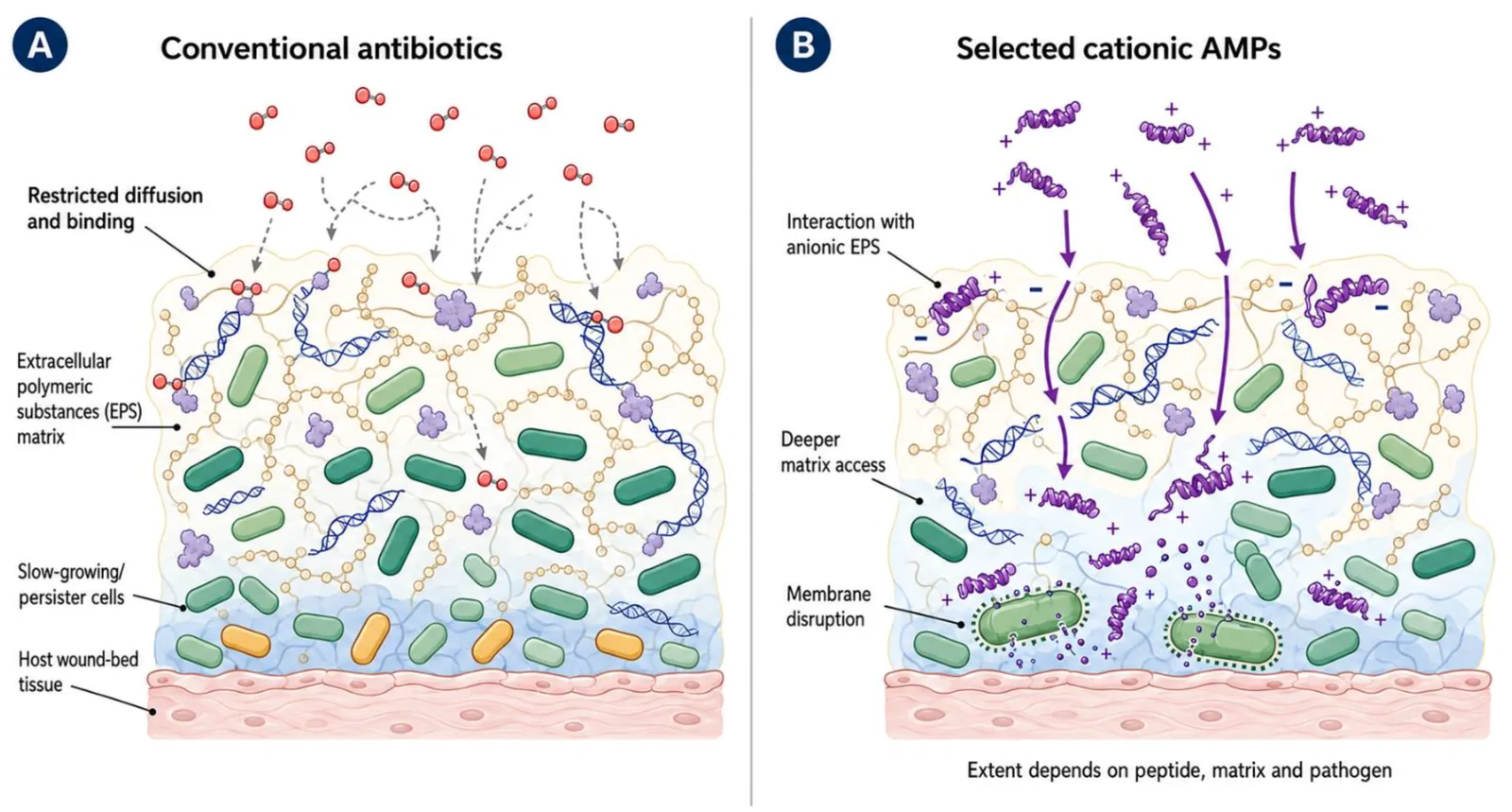
Differential interactions and penetration of conventional antibiotics and selected cationic antimicrobial peptides within mature biofilms. **(A)** Depending on their physicochemical properties, some conventional antibiotics exhibit delayed or incomplete penetration because the extracellular polymeric substances (EPS) matrix—comprising polysaccharides, proteins, extracellular DNA, lipids, and water—can retard diffusion or sequester antimicrobial molecules. Reduced metabolic activity and persister-cell formation in deeper biofilm regions further diminish antibiotic efficacy. **(B)** Selected cationic antimicrobial peptides (AMPs) interact electrostatically with anionic EPS components. Such interactions may result in matrix sequestration, whereas peptides with favorable physicochemical properties may achieve deeper access and disrupt bacterial membranes. These outcomes are peptide-, matrix-, and pathogen-dependent and should not be generalized to all AMPs.

Conventional antibiotics may show reduced activity against biofilm-associated bacteria because of limited penetration, altered bacterial metabolism, and the presence of persister cells (**Figure 8A**). Cationic AMPs can interact with anionic components of the EPS matrix and bacterial membranes (**Figure 8B**); depending on the peptide and biofilm composition, this interaction may result in either matrix sequestration or deeper biofilm penetration. Two unrelated AMP comparators illustrate general antibiofilm principles relevant to defensin development. The engineered peptides d-WRL and W(Dab)L retained activity against planktonic MRSA and established biofilms under physiologically relevant conditions (Yadav et al., 2025). Melittin demonstrated antibiofilm activity against MDR-MRSA and *P. aeruginosa*, particularly when combined with antibiotics (Mirzaei et al., 2023). These findings inform peptide optimization and combination-testing strategies but are not interpreted as defensin-specific efficacy. Nevertheless, standardized biofilm models and infection-relevant PK/PD studies remain necessary before these findings can be translated clinically (Travé-Asensio et al., 2025). Therefore, these antibiotics do not effectively kill or inhibit slow growing or persister bacterial cells; only small numbers of antibiotics can penetrate EPS. (B) Cationic AMPs can electrostatically interact with biofilm components, that are negatively charged in order to penetrate through the biofilm matrix, disrupt biofilm structure and allow for better access to bacterial membranes.

### 8.2 Synergy with conventional antibiotics

**Figure 9:**
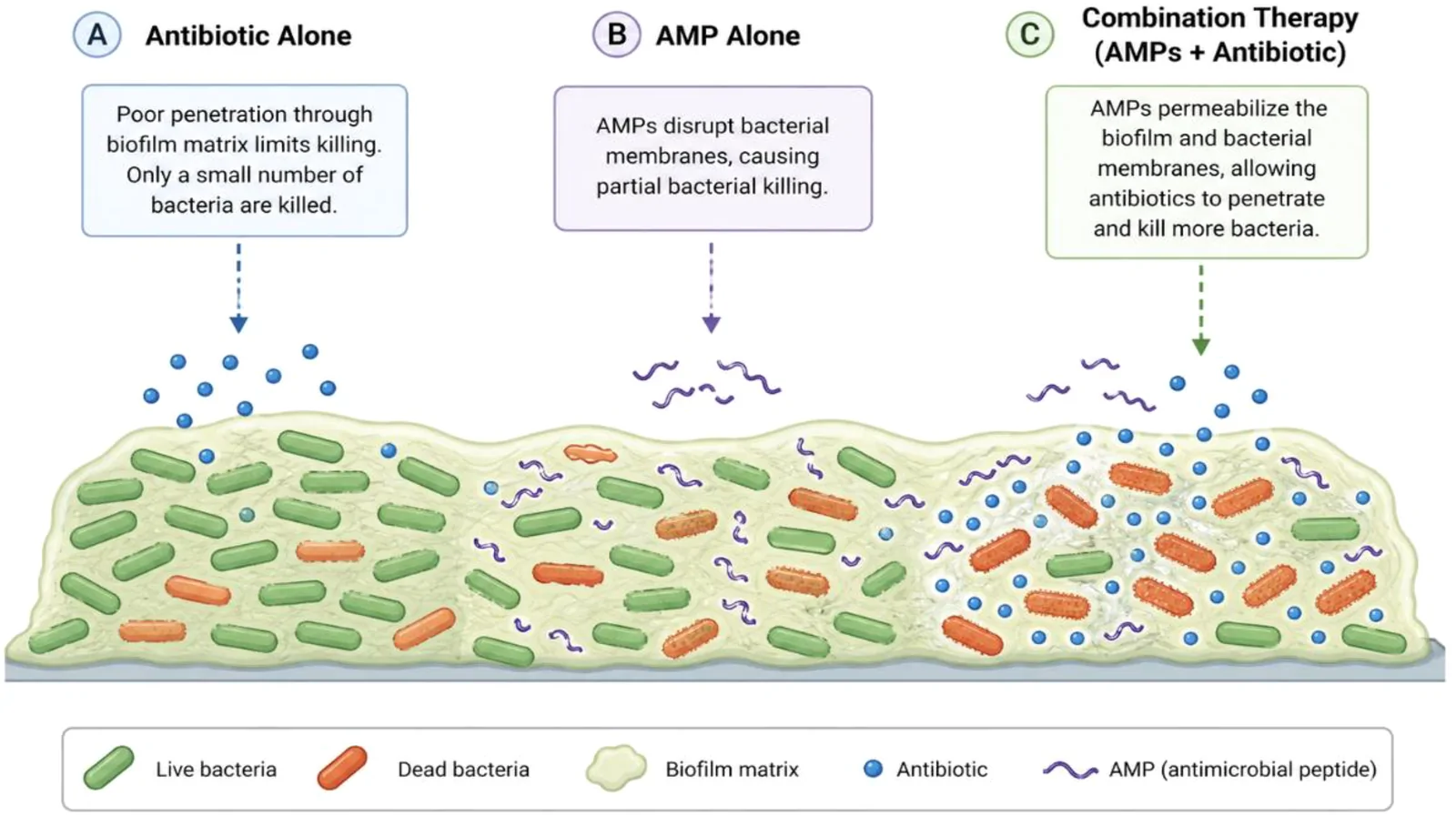
Combined use of antimicrobial peptides and conventional antibiotics against biofilm-associated infections. **(A)** Antibiotic monotherapy may show limited activity against biofilm-associated bacteria because of restricted penetration and biofilm-associated tolerance. **(B)** AMP monotherapy can disrupt bacterial membranes and may partially reduce biofilm viability. **(C)** In selected combinations, AMP-mediated membrane perturbation can enhance antibiotic penetration and bacterial killing. These effects are peptide-, antibiotic-, pathogen-, and biofilm-dependent. This schematic presents a general AMP–antibiotic combination framework and should not be interpreted as a defensin-specific or universally established mechanism.

Many AMP-antibiotic combinations show marked antibacterial synergistic effects by suppressing both planktonic growth and biofilm development. Animal studies further indicate that certain combinations improve survival outcomes and decrease infection severity. Certain AMP–antibiotic combinations can lower the effective doses of individual agents; however, any effect on resistance selection must be demonstrated for each combination rather than inferred from synergy alone. Mechanistically, AMPs often enhance membrane permeability, facilitating antibiotic entry into bacterial cells and thereby strengthening antibacterial efficacy. Although *in vitro* findings strongly support synergistic interactions between AMPs and antibiotics, clinical validation remains limited. This gap is largely due to pharmacokinetic differences and other constraints that distinguish peptides from traditional antimicrobial agents (Deo et al., 2022). Bacterial membranes are not the sole targets of AMPs, but these peptides may also interfere with cell walls or intracellular components such as nucleic acids, proteins, enzymes, and organelles, ultimately disrupting essential metabolic pathways and causing cell death. Clarifying these intracellular mechanisms is important for interpreting antibacterial activity and designing candidate-specific resistance studies. Despite their mechanistic diversity, AMPs face several challenges that limit clinical translation. Naturally derived peptides often suffer from low yield and purity, along with suboptimal pharmacokinetic properties including poor absorption, limited bioavailability, inadequate permeability, and rapid metabolism or clearance. To address these limitations, researchers have employed diverse strategies to enhance peptide performance. Approaches include modifying amino acid composition, introducing post-translational alterations, recombining peptide sequences, and utilizing computational tools for rational design and discovery. Continued research in these areas will deepen understanding of AMP mechanisms and support their development as innovative therapeutics for complex infectious diseases (Chen et al., 2023). Since biofilms show increased resistance to antibiotics, conventional antibiotics may show reduced efficacy against biofilm-associated bacteria. AMPs have the potential to enhance the activity of antibiotics by either sensitizing dormant cells, modifying the structure of biofilms, or facilitating increased penetration across the cell membrane (**Figure 9**). Melittin, an unrelated bee-venom AMP, is included solely as a combination comparator. It enhanced antibiotic activity against MDR-MRSA and *P. aeruginosa* biofilms, potentially through membrane disruption and improved antibiotic access (Mirzaei et al., 2023). This finding demonstrates a general AMP–antibiotic combination principle and is not evidence of equivalent synergy in defensins. Although defensins such as plectasin can engage complementary bacterial targets, including lipid II, antibiotic synergy must be demonstrated experimentally for each peptide–antibiotic–pathogen combination (Jekhmane et al., 2024).

### 8.3 Device-associated infections and surface coatings

Medical devices such as catheters, prosthetic joints, vascular grafts, and wound dressings are susceptible to biofilm-associated infections. Once bacteria establish an infection, it is usually much harder to treat the infection and to prevent bacterial adherence and the formation of a biofilm at the earliest possible time. Because of their amphipathic characteristics and structural stability, defensins and host-defense peptides (HDPs) can be immobilized on the surfaces of biomaterials (Travé-Asensio et al., 2025). Also, because of their cationic charge, they are able to interact with the bacterial membranes at the interface, thus potentially decreasing the initial colonization of bacteria, thus, decreasing initial bacterial adhesion and preventing subsequent biofilm formation before mature biofilms become established. AMP-based coatings are being investigated because localized surface activity may limit bacterial adhesion and biofilm establishment; however, repeated-exposure and resistance-selection studies are required for each coating–pathogen system (Xuan et al., 2023). This issue is also considered in the context of implantation sites, where there is always going to be exposure to serum and host proteases (Yadav et al., 2025). Thus, among the design factors for coatings are maintaining an appropriate amphipathic orientation after immobilization, sustaining activity in situations exposed to serum, and reduction of cytotoxicity to the host.

### 8.4 Topical and wound contexts

Chronic wounds including diabetic ulcers and burns are often characterized by high protease burdens as well as polymicrobial biofilms where topical application of medication would help to reduce systemic exposure as well as serum inactivation. The role of β-defensins in localized therapeutic use is supported by evidence that they act naturally at epithelial barriers and are documented to help maintain tissue integrity when bacteria infect tissue (Zhao et al., 2024; Tian et al., 2025). In these situations, where proteolytic degradation and physiological ionic strength result in decreased peptide efficacy, AMPs that demonstrate enhanced proteolytic stability and salt tolerance appear to be the best candidates (Yadav et al., 2025). Because topical treatment may produce repeated or prolonged peptide exposure, candidate-specific resistance and cross-resistance testing is required before suitability for repeated application can be claimed (Salehi et al., 2025). Topical and wound-directed delivery may reduce some systemic pharmacokinetic barriers for delivering defensin-based products where successful anti-biofilm activity in the context of wounds and devices require various combinations of activities to be successful including preventing bacteria from adhering to surfaces to form a biofilm, penetrating and partially disrupting the extracellular matrix of biofilms that have already formed, killing bacteria that are embedded in mature biofilms and using antibiotics in synergy with these therapies (Yadav et al., 2025; Mirzaei et al., 2023; Travé-Asensio et al., 2025, and Xuan et al., 2023). Taken together, defensins and AMPs provide an unprecedented opportunity for biofilm-targeted uses, particularly in the context of topical, wound, and device-associated infection scenarios where combination and localized delivery methods can be used to enhance therapeutic success against multidrug-resistant bacteria.

## 9 Bacterial resistance, tolerance, and cross-resistance mechanisms

Experimental evidence does not support a uniform ranking of AMP and antibiotic resistance risks. In *Escherichia coli*, experimental evolution against 14 AMPs produced lower average but markedly more heterogeneous resistance gains than evolution against 12 antibiotics; some peptides showed little change, whereas others selected substantial resistance (Spohn et al., 2019). Conversely, serial exposure of *Staphylococcus aureus* to host-defense peptides selected stable resistance with little or no detectable fitness cost and reduced susceptibility to human defense peptides and clinically used antibiotics (Kubicek-Sutherland et al., 2017). Resistance risk should therefore be defined for each peptide–pathogen combination and exposure protocol rather than inferred from membrane activity or mechanistic multiplicity. A major mechanism involves reduction of the net negative charge on the bacterial envelope. In Gram-positive bacteria, the *dltABCD* system promotes D-alanylation of teichoic acids, while *mprF* modifies membrane phospholipids by lysinylation of phosphatidylglycerol. Both processes decrease electrostatic attraction between cationic peptides and the bacterial surface, thereby reducing susceptibility to defensins and other AMPs (Assoni et al., 2020; Slavetinsky et al., 2022) This is particularly relevant to *Staphylococcus aureus*, where altered surface charge is also linked to reduced susceptibility to daptomycin, raising concern that therapeutic pressure from membrane-active agents may select phenotypes with broader tolerance to host-defense peptides and clinically used lipopeptides (Thitiananpakorn et al., 2020).

In Gram-negative bacteria, resistance frequently involves lipid A and LPS remodelling. Modification of lipid A with 4-amino-4-deoxy-L-arabinose or phosphoethanolamine, commonly regulated through systems such as PhoPQ, PmrAB, or mediated by mobile *mcr* genes, reduces the negative charge of the outer membrane and limits peptide binding (Poirel et al., 2017; Jangir et al., 2023). This mechanism is central to colistin resistance but may also confer reduced susceptibility to endogenous host-defense peptides, creating a biologically important cross-resistance risk between therapeutic polymyxins, defensin-like agents, and innate immune effectors (Jangir et al., 2023). Therefore, any defensin-based antibacterial program should test candidate peptides not only against antibiotic-susceptible strains, but also against colistin-resistant, *mcr*-positive, and lipid A–modified Gram-negative isolates.

Capsule production provides an additional extracellular defense against cationic peptides. Capsular polysaccharides can shield the bacterial surface and limit peptide interaction with the outer membrane. In *Klebsiella pneumoniae*, capsule-deficient mutants showed increased susceptibility to human neutrophil defensin-1, human β-defensin-1, lactoferrin, protamine, and polymyxin B, while greater capsule production was associated with increased peptide resistance. Exposure to polymyxin B or lactoferrin also increased capsule production and *cps* transcription, indicating that capsule-mediated protection can be inducible (Campos et al., 2004). Because capsule abundance and composition vary among strains and growth conditions, this mechanism should be evaluated directly in clinically relevant encapsulated isolates.

Proteolytic degradation represents another resistance and tolerance mechanism. Several pathogens secrete extracellular proteases that can cleave host-defense peptides before they reach their membrane targets (Joo et al., 2016). This issue is highly relevant in chronic wounds, burns, cystic fibrosis airways, and biofilm-rich infections, where bacterial and host-derived proteases are abundant. Defensin scaffolds are partly protected by disulfide bonds, and cyclic or D-amino-acid-modified analogues may further improve stability. However, protease resistance must be demonstrated experimentally under infection-relevant conditions rather than assumed from peptide structure alone.

Active efflux and inducible envelope-stress responses provide additional protection. In *Neisseria gonorrhoeae*, MtrCDE enhanced extracellular survival during exposure to human neutrophils, neutrophil extracellular traps, and degranulation products and provided protection against some, but not all, neutrophil-derived antimicrobial peptides (Handing et al., 2018). Two-component systems coordinate broader adaptive responses: PhoPQ and PmrAB regulate lipid A remodeling in Gram-negative bacteria, whereas GraRS promotes D-alanylation and related envelope adaptations in *Staphylococcus aureus*. Deletion of *graRS* increased bacterial surface negativity and susceptibility to LL-37 and neutrophil-mediated killing, demonstrating that peptide resistance can be actively induced through stress sensing and regulatory control (Kraus et al., 2008; Poirel et al., 2017).

Biofilm growth produces predominantly phenotypic tolerance rather than a single heritable resistance mechanism. The extracellular polymeric substance can bind or retard cationic peptides, while slow growth, metabolic heterogeneity, persister cells, high cell density, and locally elevated protease activity further reduce peptide killing. Consequently, susceptibility measured in planktonic cultures may not predict activity against mature biofilms. Defensin candidates intended for biofilm-associated infections should therefore be evaluated using biofilm-specific endpoints, including minimum biofilm inhibitory and eradication concentrations, penetration assays, and time-kill analysis (Joo et al., 2016; Tajer et al., 2024).

Because many of these adaptations modify general envelope properties rather than peptide-specific targets, they may protect bacteria against both therapeutic peptides and endogenous defensins or cathelicidins. Selection by defensin-like therapeutics could therefore reduce bacterial susceptibility to peptides produced by neutrophils, epithelial cells, and mucosal surfaces, potentially weakening innate immune clearance (Assoni et al., 2020; Jangir et al., 2023). This risk is not identical to conventional antibiotic resistance, but it is translationally important. Experimental and clinical evidence shows that resistance to membrane-active peptide antibiotics, particularly colistin, can sometimes increase tolerance to host antimicrobial peptides such as LL-37, lysozyme, and defensins, although this effect is species-, mechanism-, and context-dependent. Selection of peptide-tolerant bacteria during *in vitro* development or repeated topical exposure may therefore favor strains with improved survival against mucosal innate immunity, especially when resistance arises through broad envelope remodeling rather than a narrow drug-specific target (Napier et al., 2013). For this reason, resistance assessment should become a standard component of defensin development. Future studies should combine serial-passage selection, cross-resistance testing against human defensins and cathelicidins, whole-genome sequencing of evolved mutants, surface-charge assays, protease-susceptibility testing, and evaluation against strains carrying relevant envelope-modifying alterations, including *dlt*, *mprF*, *pmrAB, phoPQ*, *mgrB*, and *mcr*. Combination therapy may reduce resistance emergence, but this should be demonstrated experimentally rather than assumed (Makarova et al., 2018). Thus, the key translational question is whether the frequency, magnitude, stability, and cross-resistance consequences of resistance can be acceptably limited for a specific defensin scaffold without compromising host innate immune function (**Table 4**).

**Table 4:** Bacterial mechanisms that reduce susceptibility to defensins and other host-defense peptides and their potential cross-resistance implications.

Bacterial adaptation	Molecular mechanism	Key genes/regulatory systems	Effect on defensin/HDP activity	Potential cross-resistance implication	References
Gram-positive surface-charge modification	D-alanylation reduces cell-envelope negativity	*dltABCD*	Decreases peptide binding	May reduce susceptibility to cationic peptides; species- and compound-dependent	Assoni et al., 2020
Membrane phospholipid modification	MprF produces and translocates lysyl-PG, reducing membrane anionicity	*mprF*; MprF	Limits peptide binding, insertion, and membrane damage	May affect HDPs and daptomycin; variant- and compound-dependent	Slavetinsky et al., 2022; Thitiananpakorn et al., 2020
Gram-negative LPS/lipid A remodeling	Addition of L-Ara4N or phosphoethanolamine lowers lipid A negativity	PhoPQ, PmrAB; *mgrB*, *arnBCADTEF*, *mcr*	Reduces outer-membrane binding and disruption	May confer cross-resistance to colistin and some HDPs; peptide- and strain-dependent	Poirel et al., 2017; Jangir et al., 2023
Capsule production	Capsule shields the surface and sequesters peptides	Species-specific *cps* loci	Restricts peptide access to membrane targets	May protect against multiple peptides; capsule-, species-, and peptide-dependent	Fleeman et al., 2020
Proteolytic degradation	Proteases cleave susceptible peptides	*aur*, *lasB*, and other protease loci	Inactivates peptides before target engagement	May affect peptides sharing protease-sensitive sequences	Tajer et al., 2024
Efflux- or transport-mediated reduced susceptibility	Transport systems lower peptide accumulation	*mtrCDE*; MtrCDE	Reduces peptide concentration at target sites	May affect therapeutic and host-derived peptide substrates	Handing et al., 2018
Envelope-stress and peptide-sensing responses	Peptide sensing activates coordinated envelope remodeling	PhoPQ, PmrAB, GraRS–VraFG	Reduces peptide binding and membrane damage	May provide context-dependent protection against peptides and envelope-active antibiotics	Kawada-Matsuo et al., 2021; Tajer et al., 2024
Biofilm-associated tolerance	EPS sequestration, poor diffusion, metabolic heterogeneity, and persisters	Multigenic biofilm and persistence networks	Lowers free peptide levels and delays killing	Mainly reversible phenotypic tolerance to peptides and antibiotics	Tajer et al., 2024; Xuan et al., 2023
Therapeutic peptide–HDP cross-resistance	Shared envelope-remodeling mechanisms reduce susceptibility to diverse cationic peptides	*mprF*, *dltABCD*, *mcr*, and regulatory loci	Reduces activity of defensins, cathelicidins, and related HDPs	Experimentally demonstrated but not universal; mechanism- and peptide-dependent	Kubicek-Sutherland et al., 2017; Jangir et al., 2023

## 10 Translational pathways for defensin-based therapeutics

### 10.1 Engineered defensin analogues

The therapeutic value of defensins is unlikely to depend on direct clinical use of native peptides alone. Native defensins provide biologically validated scaffolds, but their translation is limited by proteolytic exposure, variable activity under physiological salt and serum conditions, manufacturing complexity, and possible host-cell toxicity at higher concentrations. Therefore, the most realistic strategy is to redesign defensin scaffolds while preserving the structural features responsible for selective microbial targeting. Recent peptide-drug development has benefited from improved synthesis, sequence modification, structural optimization, and delivery technologies, all of which are relevant to defensin-derived therapeutics (Wang et al., 2022).

Engineering approaches include sequence truncation, residue substitution, cyclization, disulfide stabilization, D-amino acid incorporation, and rational modulation of amphipathicity. These modifications aim to improve protease resistance, reduce nonspecific serum binding, enhance tissue residence, and widen the therapeutic window. However, optimization should not be guided by antimicrobial potency alone. A defensin analogue with a low MIC but poor stability, high hemolysis, or strong serum sequestration is unlikely to progress clinically. For this reason, engineered defensins should be evaluated using a combined profile of antimicrobial activity, salt tolerance, serum stability, protease resistance, cytotoxicity, antibiofilm performance, and resistance selection potential. Recent reviews of human β-defensins and synthetic analogues emphasize that clinical translation still depends on overcoming stability, bioavailability, resistance, and toxicity barriers, rather than simply improving *in vitro* activity (Chen et al., 2024).

### 10.2 Defensin mimetics and small-molecule scaffolds

Defensin mimetics represent a more drug-like route for preserving the functional logic of defensins while avoiding some liabilities of natural peptides. These compounds are designed to reproduce key physicochemical features of host-defense peptides, particularly spatially organized cationic and hydrophobic domains, but with improved chemical stability, lower manufacturing burden, and potentially better pharmacokinetic control. Brilacidin is the best-known example of a non-peptidic defensin mimetic; it has been described as a small molecule designed to mimic host-defense peptide activity and has been evaluated in clinical contexts including acute bacterial skin and skin-structure infections (Mensa et al., 2014).

The main advantage of defensin mimetics is not that they are “stronger defensins,” but that they convert host-defense peptide principles into chemically tractable therapeutic formats. This may allow improved formulation, topical or systemic delivery, and reduced susceptibility to proteolysis. Nevertheless, mimetics should not be treated as a uniform class. Their mechanisms may include membrane depolarization, envelope disruption, intracellular effects, or immunomodulatory activity depending on compound chemistry and pathogen context. Therefore, each mimetic requires compound-specific validation of mechanism, toxicity, resistance risk, and host-cell compatibility.

### 10.3 Delivery strategies

Delivery remains one of the central barriers separating defensin activity *in vitro* from therapeutic performance *in vivo*. Systemic administration may expose peptides to serum proteins, proteases, renal clearance, and off-target host membranes, whereas topical, wound-directed, inhaled, or device-associated delivery may better match the biological niches where defensins naturally function. Drug-delivery systems can improve AMP stability, bioavailability, local retention, and controlled release; recent analyses highlight nanoparticles, hydrogels, lipid-based systems, and self-assembled peptide platforms as major approaches for improving AMP translation (Zheng et al., 2025).

For defensin-based therapeutics, delivery design should be indication-specific. Hydrogels and nanoparticle–hydrogel hybrids are particularly attractive for wounds, burns, diabetic ulcers, and device-associated infections because they can provide localized release and reduce systemic exposure. Nanoparticle–hydrogel systems have been reviewed as controlled and targeted antimicrobial delivery platforms, including for antimicrobial peptides (Ahmad et al., 2024). Lipid-based and polymeric systems may be more relevant for mucosal or systemic delivery, but they must be assessed for peptide release kinetics, tissue penetration, inflammatory effects, and compatibility with biofilm environments. In this context, delivery is not simply a formulation step; it is part of the therapeutic mechanism.

### 10.4 Combination therapy logic

Defensins and defensin-like agents are best positioned as components of combination therapy rather than universal replacements for antibiotics. Their membrane-active or envelope-targeting effects can increase bacterial permeability, weaken biofilm structure, and enhance antibiotic access to intracellular targets. This provides a mechanistic basis for combining defensin-based agents with cell-wall inhibitors, aminoglycosides, fluoroquinolones, or anti-biofilm strategies, depending on pathogen and infection site. Clinical-trial reviews of AMPs emphasize that only a limited number have reached clinical use despite strong preclinical promise, partly because potency alone does not overcome pharmacokinetic, toxicity, and trial-design barriers (Dijksteel et al., 2021). Combination therapy should therefore be framed carefully. Synergy should be demonstrated using standardized checkerboard assays, time-kill kinetics, biofilm models, resistance-selection experiments, and, where possible, animal infection models. A combination that reduces MIC values in planktonic culture may fail in serum, biofilm, wound exudate, or high-salt environments. The strongest translational logic is not simply “defensin plus antibiotic,” but mechanism-matched pairing: a peptide or mimetic that disrupts envelope integrity should be paired with an antibiotic whose intracellular or cell-wall access is limiting.

### 10.5 Safety and immunomodulation balance

Safety is a decisive translational filter for defensin-based therapeutics. Defensins are not merely antimicrobial molecules; they can influence cytokine signaling, leukocyte recruitment, epithelial repair, wound healing, and inflammatory tone. This dual activity is beneficial when it supports pathogen clearance and tissue repair, but it can become problematic if engineered analogues amplify inflammation, damage host membranes, or interfere with immune homeostasis. Reviews of host-defense peptides emphasize that their therapeutic value lies in both antimicrobial and immunomodulatory activity, but this duality also requires careful control of dose, route, tissue exposure, and inflammatory context (Drayton et al., 2021).

**Figure 10:**
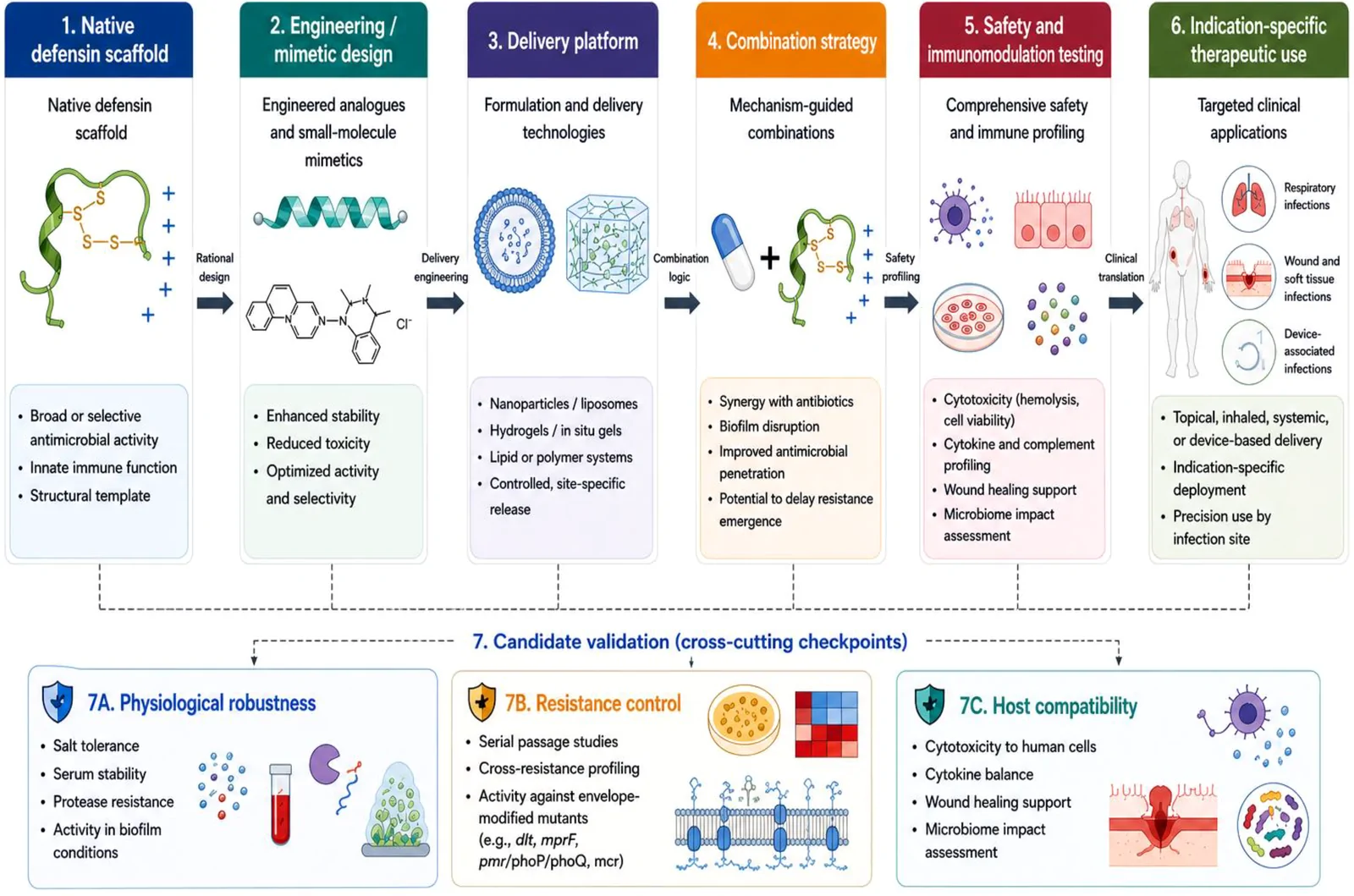
Translational roadmap for defensin-based therapeutics: from scaffold engineering to clinical application. This figure summarizes the translational pathway for defensin-based therapeutics, from native scaffolds to clinical use. It highlights engineering, mimetic design, delivery platforms, combination strategies, and safety testing, with candidate selection guided by physiological robustness, resistance-risk characterization, and host compatibility.

For clinical development, safety evaluation should move beyond simple antimicrobial potency to include hemolysis, epithelial and immune-cell cytotoxicity, cytokine profiling, complement-related inflammatory responses, wound-healing compatibility, microbiome effects, and repeated-exposure testing. The aim is not to suppress immunomodulation, which may contribute to therapeutic benefit, but to define a safe and controllable host-response window. Defensin-based therapeutics will be most persuasive when antimicrobial efficacy, resistance-selection data, tissue compatibility, and regulated immune modulation are demonstrated together (Hancock et al., 2016). The translational future of defensins depends on moving beyond the simple view of these peptides as natural antimicrobials. Their strongest value lies in using defensin scaffolds as templates for engineered analogues, defensin mimetics as more drug-like surrogates, delivery systems as activity-preserving platforms, and combination therapy as a resistance-conscious strategy. The clinically relevant question is not whether defensins kill MDR bacteria *in vitro*, but whether defensin-derived therapeutics can retain activity under physiological conditions while remaining safe, manufacturable, and compatible with host immunity (Fu et al., 2023). The translational framework and key decision points guiding defensin-based therapeutic development are summarized in **Figure 10**.

## 11 Future perspectives: translating defensins into resistance-aware anti-MDR therapeutics

Future defensin development should follow an indication-specific, stepwise framework. Candidates should first demonstrate reproducible activity under physiologically relevant conditions, followed by adequate infection-site exposure, safety, and efficacy in infection-matched models. **Table 5** summarizes the assays, models, PK/PD endpoints, and safety criteria proposed for each stage. Because defensins differ in mechanism, formulation, and route, advancement criteria should be defined for each candidate rather than applied uniformly across the class.

**Table 5:** Proposed stage-specific assessment framework for defensin-based therapeutic development.

Priority	Minimum assessment	Core endpoints	Target setting
Antibacterial activity	Reference BMD, MBC, time-kill, MDR clinical isolates	MIC distribution, MBC/MIC, log_10_ killing, regrowth	All candidates
Physiological robustness	Salt/pH, serum, proteases, mature biofilms	MIC shift, peptide stability, MBIC/MBEC, biofilm killing	All candidates
PK/PD	Protein binding, plasma and infection-site exposure, dose fractionation	fAUC_0_–_24_/MIC, fCmax/MIC, %fT>MIC, half-life, PTA	Route-specific development
Safety	Hemolysis, primary-cell cytotoxicity, cytokines/complement, repeat-dose toxicity	HC_50_, CC_50_, selectivity index, NOAEL, safety margin	Route- and site-specific
Infection models	Infected wound, catheter/implant biofilm, or matched mucosal model	Bacterial burden, eradication, recurrence, healing/barrier integrity	Wound, device, and mucosal infections
Delivery	Release, peptide recovery, protease protection, tissue retention	Release kinetics, intact-peptide exposure, local tolerability	Indication-specific formulation
AI optimization	Multi-objective design with prospective experimental validation	Improvement over the parent peptide in activity, stability, or selectivity	Candidate prioritization
Resistance and stewardship	Resistance frequency, serial passage, cross-resistance, WGS	MIC shift, resistance stability, fitness cost, mutation profile	Restricted, susceptibility-guided use

AUC, area under the concentration–time curve; CC_50_, concentration reducing cell viability by 50%; HC_50_, concentration causing 50% hemolysis; MBC, minimum bactericidal concentration; MBEC, minimum biofilm eradication concentration; MBIC, minimum biofilm inhibitory concentration; PK/PD, pharmacokinetics/pharmacodynamics.

First, susceptibility testing should begin with CLSI M07 reference broth microdilution and include MBC, time-kill analysis, and an adequate panel of MDR clinical isolates. Assay medium, inoculum, peptide concentration, and quality-control strains should be reported. Candidates should then be retested under physiological salt, serum, protease-rich, and mature-biofilm conditions, with MIC shifts, killing kinetics, and MBIC/MBEC used to assess retained activity (CLSI, 2024; Zheng et al., 2025). Second, PK/PD should be addressed before late-stage development. Studies should quantify intact peptide in plasma and, where feasible, at the infection site, together with protein binding and tissue distribution. AUC_0_–_24_, Cmax, half-life, clearance, and fraction unbound should be related to MIC through fAUC_0_–_24_/MIC, fCmax/MIC, and %fT>MIC. Dose-fractionation and probability-of-target-attainment analyses should then define the exposure required for efficacy (Ting et al., 2020; Dijksteel et al., 2021). Safety should be treated as an independent advancement criterion. Initial assessment should combine hemolysis in human erythrocytes with cytotoxicity in route-relevant mammalian cells and report HC_50_, CC_50_, and the selectivity index. Local candidates require irritation and tissue-compatibility testing, whereas systemic or repeated dosing should include hematology, clinical chemistry, organ histopathology, and immunogenicity. Safety findings should be interpreted against the expected therapeutic exposure because *in vitro* hemolysis alone does not reliably predict *in vivo* toxicity (Greco et al., 2020; Wang et al., 2022). Third, development should be organized around a defined clinical setting. Local indications are the most realistic starting point, particularly chronic wounds, device-associated biofilms, and selected mucosal infections. Wound candidates should be assessed in ex vivo skin and infected-wound models; device candidates in flow-cell biofilms and catheter or implant models; and mucosal candidates in differentiated epithelial cultures followed by an anatomically matched infection model. Core outcomes should include bacterial burden, biofilm eradication, recurrence, tissue healing or barrier integrity, and local peptide exposure (Grassi et al., 2019; Gera et al., 2022).

Fourth, combination therapy should be designed based on mechanism, not trial and error. Defensins and their mimetics may work best as membrane-active agents that improve antibiotic entry into bacterial cells or biofilms. This supports pairing them with antibiotics limited by poor penetration. Such combinations may lower antibiotic doses and help restore activity against resistant pathogens (Pletzer and Hancock, 2016). However, synergy should be demonstrated under physiologically relevant conditions, including serum and biofilm models, rather than only in conventional planktonic assays.

Fifth, resistance must be treated as a candidate-specific design parameter. Because resistance outcomes vary across peptides, pathogens, and experimental systems, defensin development should include serial-passage experiments, resistance-frequency measurements, cross-resistance testing against endogenous host-defense peptides and conventional antibiotics, and genomic analysis of adapted populations. This is particularly important because peptide exposure can select stable resistance with limited fitness cost and reduced susceptibility to innate immune effectors (Kubicek-Sutherland et al., 2017). Defensin development should therefore include serial-passage testing, cross-resistance assessment, and genomic analysis of bacterial adaptation. This is essential because new antimicrobials must be tested against resistance development, not assumed to remain effective over time (Laxminarayan et al., 2013; Murray et al., 2022).

Sixth, AI should support multi-objective candidate selection rather than potency prediction alone. Models should consider antimicrobial activity, serum and protease stability, biofilm activity, cytotoxicity, hemolysis, solubility, and manufacturability. Selected peptides must then undergo prospective experimental validation. Delivery systems should similarly demonstrate improved peptide stability, controlled release, infection-site exposure, or local tolerability relative to the unformulated peptide (Das et al., 2021; Gera et al., 2022; Pimentel et al., 2026). Finally, stewardship should be defined before clinical use. Each candidate requires a restricted pathogen–indication profile, a standardized susceptibility method, and a predefined role as monotherapy or adjunctive treatment. Baseline and post-treatment isolates should be retained to monitor resistance and cross-resistance. Initial use should focus on microbiologically confirmed MDR infections for which available therapies are inadequate (O’Neill, 2016; Murray et al., 2022). Therefore, defensin-based therapeutics must be developed within resistance-conscious frameworks that balance efficacy with long-term sustainability.

**Figure 11:**
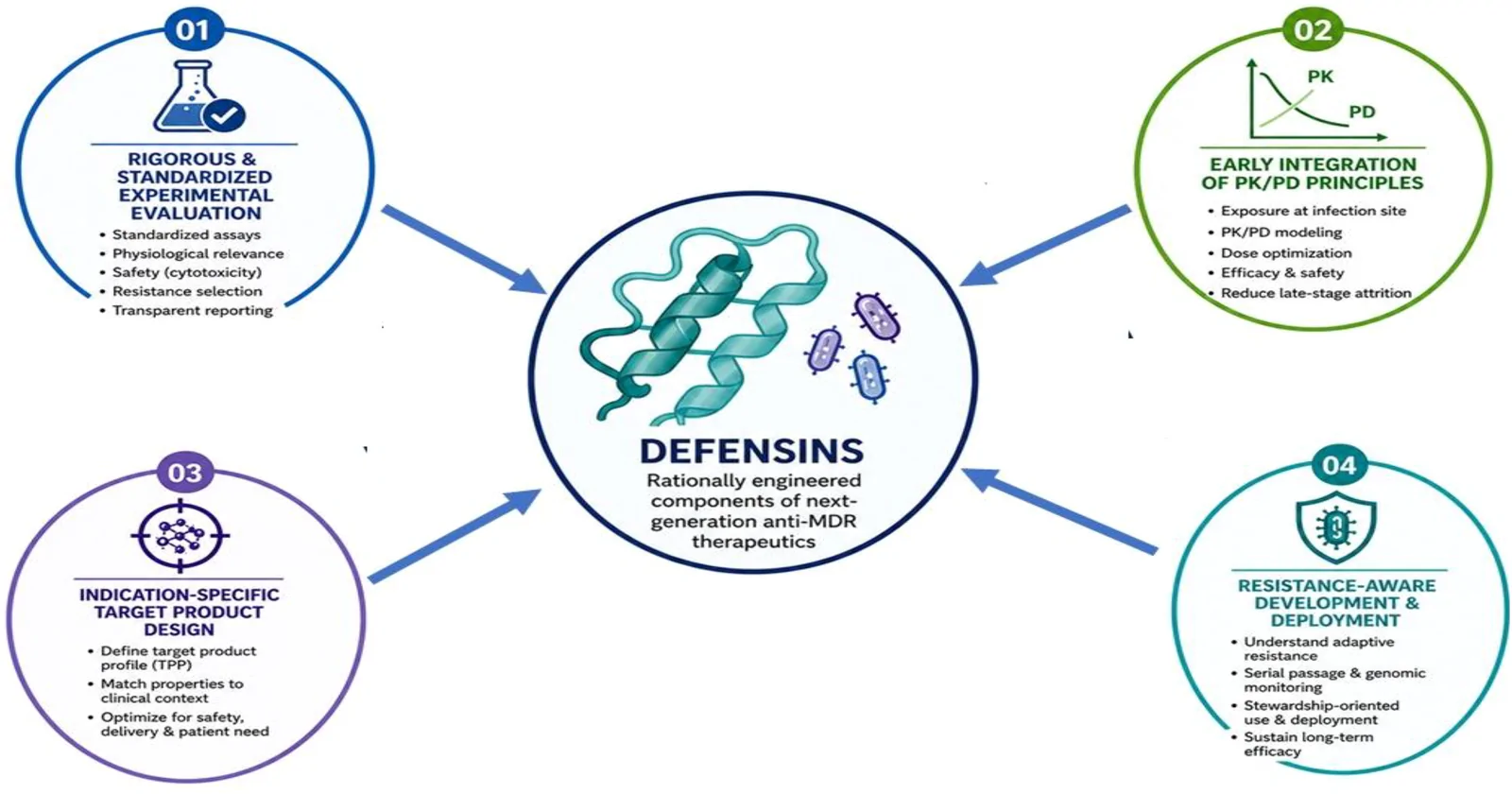
Translational roadmap for defensin-based anti-MDR therapeutics. This schematic shows the four main pillars for translating defensins into anti-MDR therapeutics: standardized testing, early PK/PD assessment, indication-specific design, and resistance-aware use. Together with AI-guided optimization and advanced delivery, these pillars can help move defensins from laboratory activity toward clinical application.

In summary, the translational success of defensins depends on four non-negotiable pillars: (i) rigorous and standardized experimental evaluation, (ii) early integration of PK/PD principles, (iii) indication-specific target product design, and (iv) resistance-aware development and deployment. When combined with AI-assisted optimization and advanced delivery technologies, these principles position defensins not as empirical natural antimicrobials, but as rationally engineered components of next-generation anti-MDR therapeutic strategies. The translational framework guiding defensin-based therapeutic development is summarized in **Figure 11**, highlighting the four non-negotiable pillars required for clinically relevant anti-MDR applications.

## 12 Conclusion

Defensins represent one of the most biologically persuasive antimicrobial peptide families for anti-MDR drug discovery, but their value lies less in direct use as native molecules and more in their function as structured, engineerable therapeutic scaffolds. Their conserved disulfide-rich architecture, cationic and amphipathic surfaces, immunomodulatory capacity, and ability to act through membrane disruption, lipid II binding, biofilm interference, intracellular effects, or pathogen entrapment provide a strong mechanistic basis for therapeutic development. However, this promise must be interpreted with caution. Defensins are not inherently resistance-proof, and bacterial adaptation through envelope remodeling, proteolysis, efflux, biofilm tolerance, and cross-resistance to host-defense peptides remains a real translational concern.

The most realistic path forward is therefore not to position defensins as universal replacements for antibiotics, but as rationally engineered components of targeted anti-MDR strategies. Future studies should implement the priorities summarized in **Table 4** by linking standardized antimicrobial testing with infection-site PK/PD, route-appropriate safety assessment, and efficacy in indication-matched models. AI-guided optimization and delivery platforms should be evaluated through measurable improvements in these endpoints, while resistance testing and surveillance should accompany development and eventual clinical use. The main limitation of the current evidence is that much of the defensin literature remains preclinical, heterogeneous, and heavily dependent on *in vitro* assays that do not fully reproduce infection-site complexity, host immunity, pharmacokinetics, or long-term resistance dynamics. Accordingly, defensin-based therapeutics will become clinically credible only when antimicrobial potency, physiological robustness, safety, manufacturability, candidate-specific resistance and cross-resistance risks, and clinical-use strategy are demonstrated together.
